# Discovery of candidate antibodies and antigens for acute myeloid leukemia therapy by combining whole-cell phage display selection and CRISPR-Cas9 library screening

**DOI:** 10.1093/abt/tbag043

**Published:** 2026-09-12

**Authors:** Koji Hashimoto, Verena Konetzki, Andoni Garitano-Trojaola, Sabrina Kraus, Sabrina R Friedel, Michael Hudecek, Haiyong Peng, Christoph Rader

**Affiliations:** Department of Life Sciences, Graduate School of Arts and Sciences, The University of Tokyo, Tokyo, 153-8902, Japan; Department of Immunology and Microbiology, The Herbert Wertheim UF Scripps Institute for Biomedical Innovation & Technology, University of Florida, Jupiter, FL 33458, United States; Department of Immunology and Microbiology, The Herbert Wertheim UF Scripps Institute for Biomedical Innovation & Technology, University of Florida, Jupiter, FL 33458, United States; Chair for Cellular Immunotherapy, Department of Internal Medicine II, University Hospital Würzburg, 97080 Würzburg, Germany; Chair for Cellular Immunotherapy, Department of Internal Medicine II, University Hospital Würzburg, 97080 Würzburg, Germany; Department of Internal Medicine II, University Hospital Würzburg, 97080 Würzburg, Germany; Chair for Cellular Immunotherapy, Department of Internal Medicine II, University Hospital Würzburg, 97080 Würzburg, Germany; Chair for Cellular Immunotherapy, Department of Internal Medicine II, University Hospital Würzburg, 97080 Würzburg, Germany; Fraunhofer Institute for Cell Therapy and Immunology (IZI), Leipzig & Branch Site Cellular Immunotherapy, 97070 Würzburg, Germany; National Center for Tumor Diseases (NCT), Site Würzburg-Erlangen-Regensburg-Augsburg (WERA), 97080 Würzburg, Germany; Bavarian Center for Cancer Research (BZKF), Lighthouse Cellular Immunotherapies, 97080 Würzburg, Germany; Department of Immunology and Microbiology, The Herbert Wertheim UF Scripps Institute for Biomedical Innovation & Technology, University of Florida, Jupiter, FL 33458, United States; Department of Immunology and Microbiology, The Herbert Wertheim UF Scripps Institute for Biomedical Innovation & Technology, University of Florida, Jupiter, FL 33458, United States

**Keywords:** acute myeloid leukemia, cancer immunotherapy, antibody discovery, phage display, CAR-T, CRISPR-Cas9 library screening

## Abstract

**Background:**

Acute myeloid leukemia (AML) remains challenging to treat and often requires intensive chemotherapy. In contrast to other hematologic malignancies, the development of effective antibody-based and cellular immunotherapies for AML has been limited by the scarcity of suitable target antigens.

**Methods:**

We applied a phage display-based whole-cell panning method in which Fab-phage were biotinylated and captured, followed by next-generation sequencing (NGS), bioinformatic, and statistical analyses. Target deconvolution was performed using a CRISPR-Cas9 knockout library, fluorescence-activated cell sorting of antigen-negative cells, and NGS-based gRNA analysis. Selected candidates were further evaluated using primary AML patient cells and chimeric antigen receptor (CAR)-T cell assays.

**Results:**

We identified 28 unique monoclonal antibodies that preferentially bound AML cell lines. CRISPR-Cas9-based target deconvolution enabled efficient identification of three cognate antigens. Selected lead candidates were validated by staining primary cells from AML patients and were engineered into CAR constructs. CAR-T cells targeting the identified antigens mediated efficient eradication of AML cell lines and primary AML cells.

**Conclusions:**

This integrated antibody-based antigen discovery and validation approach may accelerate the development of monoclonal antibody- and CAR-based immunotherapies for AML and other indications.

## Teaser

This study integrates whole-cell phage display selection with CRISPR-Cas9-based target deconvolution to identify AML-selective antibodies and their cognate antigens. The resulting antibody–antigen pairs, including candidates evaluated in CAR-T cell formats, provide a framework for accelerating immunotherapy target discovery.

## Introduction

Acute myeloid leukemia (AML) is a type of cancer that affects the bone marrow (BM) and blood. It is characterized by the rapid growth of abnormal myeloid cells, which are immature blood cells that normally develop into white blood cells, red blood cells, and platelets [1]. Although most AML patients go into remission after intensive chemotherapy, relapse is frequent, associated with poor prognosis, and often occurs due to the resistance of leukemia stem cells (LSCs) [2]. Compared with other hematologic malignancies, immunotherapeutic approaches such as antibody-based therapies and chimeric antigen receptor (CAR)-T cell therapy have shown limited success in AML, partly due to its pronounced cellular and molecular heterogeneity [3]. Only one antibody-based cancer therapy is currently approved for AML by the US Food and Drug Administration (FDA); antibody-drug conjugate (ADC) gemtuzumab ozogamicin (Mylotarg®), an anti-CD33 monoclonal antibody (mAb) conjugated to calicheamicin, is available for the treatment of relapsed or refractory AML [4]. In the case of CAR-T cell therapy, multiple targets, including CD33, CD123, CD44v6, FLT-3, CD70, CLL-1, and Siglec6, have been under investigation; however, none of these candidates have received FDA approval to date [5]. Identifying suitable targets for AML-directed immunotherapy remains challenging because most candidate antigens are not exclusively expressed on AML blasts and LSCs. Instead, they are also present on healthy hematopoietic stem and progenitor cells (HSPC), thereby increasing the risk of significant on-target/off-tumor toxicities [6–8]. Importantly, these limitations do not diminish the therapeutic relevance of established AML-associated antigens such as CD33, CD123, and FLT3. Rather, they underscore the need to identify additional and complementary targets. The heterogeneous expression of these antigens among patients and across leukemic subpopulations may limit patient coverage and facilitate the persistence or emergence of antigen-low or antigen-negative cells. Expanding the repertoire of targetable cell-surface antigens may, therefore, support the development of alternative, sequential, or combinatorial immunotherapeutic strategies and help reduce the risk of antigen escape. Accordingly, the identification of novel AML-associated surface antigens with favorable expression profiles remains an important objective in the development of more effective immunotherapies for AML.

To find new candidate antigens for cancer immunotherapy, whole-cell panning (WCP) is a powerful tool [9, 10]. Briefly, a phage display library displaying an antibody fragment is incubated with the target cancer cell lines. Cell-binding antibodies along with their genes are recovered, validated, and used for cell surface antigen deconvolution. Compared to conventional antibody selection and screening methods with defined purified proteins, WCP holds the promise of not only selecting antibodies to annotated membrane proteins but also uncovering antibodies that target cancer cell-specific isoforms, conformations [11], multiprotein complexes, glycosylations [12], or surface RNAs [13]. Thus, the antigen-agnostic outset opens possibilities for significant biological discoveries.

In previous studies, we optimized WCP for a Fab-displayed phage library. In the development process, the issue of “bald” phage, which does not display Fab, nonspecifically sticking to cells emerged as a challenge unique to applying phage display to WCP. To eliminate bald phage, we introduced a more effective approach for the selection of Fab-phage libraries on whole cells, known as Fab-phage biotinylation and capture (FBC) [14]. In the FBC method, we utilized Sortase A to biotinylate only Fab-displaying phage, facilitating their capture by streptavidin-coated magnetic beads and ultimately allowing the removal of bald phage. This modification significantly enhanced the efficiency of WCP.

Recently, we introduced elements of next-generation sequencing (NGS), bioinformatics, and statistical analysis into FBC and developed a streamlined method named FBC-seq [15]. In FBC-seq, FBC selection is carried out not only on cancer cells but also in parallel on healthy cells, such as primary peripheral blood mononuclear cells (PBMCs). Each WCP output is pooled and comprehensively read by multiplexed NGS and is used for differential abundance analysis. Antibody genes that are statistically enriched on cancer cells versus healthy cells are pursued as candidates for specific targeting and used for antigen discovery. In the current study, in an effort to conduct integrated antibody drug and target discovery for AML, we chose AML cell line Kasumi-1 as target cells and primary human PBMCs as nontarget cells. FBC-seq delivered 28 unique Kasumi-1-binding antibodies, most of which bound additional AML cell lines.

Although FBC-seq has made great progress in antibody discovery, there is still room for improvement in antigen discovery. A well-known method for antigen deconvolution is immunoprecipitation (IP); antibodies are incubated with cell lysates to form antibody–antigen complexes, which are then isolated by SDS-PAGE, for example, and antigens are identified by mass spectrometry (MS). This approach works occasionally, but it often fails because antibody–antigen complexes or the antigens themselves can be unstable, and because of the high background of co-immunoprecipitating proteins. In our previous FBC-seq study, we were not able to identify the antigens with IP/MS alone [15]. We used a combination of proteomics and transcriptomics to identify the antigens, but realized the need to more robustly and easily deconvolute antigens from antibodies. In the current study, a strategy combining an antigen knockout library and a fluorescence-activated cell sorting (FACS) method is reported. The rationale is that the cells in which the gene encoding the antigen recognized by the antibody has been knocked out are not stained with the antibody. In other words, within an antigen knockout library, isolating the cells not bound by the antibody should lead to the identification of the potential antigen. We further turned our attention to a CRISPR-Cas9 library as a knockout resource, anticipating that we could pinpoint the antigen by scrutinizing the gRNA obtained from the enriched cell population [16]. As envisioned, the antibodies obtained via FBC-seq were successfully deconvoluted by CRISPR-Cas9 library FACS and led to the identification of antigens highly expressed in AML cell lines. In summary, the workflow from antibody discovery using FBC-seq to antigen discovery via CRISPR-Cas9 library is broadly generalizable and thus facilitates the rapid advancement of therapeutic antibody candidates against otherwise unknown antigens. To demonstrate the clinical relevance of our approach, we generated Fc-silenced IgG1 and used them to stain primary leukemic cells from AML patients. Lead Fab candidates were also reformatted as scFvs for CAR-T cells, which exhibited robust anti-tumor activity in AML cell lines and primary cells, highlighting the translational potential of our antibody-based antigen discovery.

## Materials and methods

### Cell lines and primary cells

Kasumi-1 and U937 cell lines were purchased from ATCC. K562, TF-1, SKNO-1, MV4-11, HEL, and Molm-13 were generously provided by the Nimer laboratory at the University of Miami Sylvester Comprehensive Cancer Center. K562 and all AML cell lines except for TF-1 and SKNO-1 were cultured at 37°C, 5% CO_2_ in RPMI-1640 medium, supplemented with 10% FBS and 1× penicillin–streptomycin. For TF-1 and SKNO-1 culture, GM-CSF was added to the medium to achieve a final concentration of 10 ng/μL. PBMCs used for FBC selection were freshly prepared from whole human blood (OneBlood) using Ficoll (Cytiva). CD34+ HSCs were bought from STEMCELL Technologies. For the generation of human CAR T cells, healthy donor blood samples were obtained from leukocyte reduction chambers provided by the Institute for Transfusion Medicine of the University Hospital Würzburg. Primary leukemic cells from AML patients were obtained at the University Hospital Würzburg under approval by the Ethics Committee of the University of Würzburg. Ethics approval and informed consent are described in the Ethics and Consent statement.

### Antigen and antibody proteins

Gene fragments encoding the human PTPRG extracellular domain (hPTPRG-ECD; amino acids 20–736), N terminus (hPTPRG-N; amino acids 20–448), and C terminus (hPTPRG-C; amino acids 449–736), as well as the mouse PTPRG ECD (mPTPRG-ECD; amino acids 20–733), were synthesized and fused to human Fcγ1 using a short GS linker and a mutated IgG1 hinge sequence (GGGGSEPKSSDKTHTCPPCP, with the underlined C-to-S mutation) after custom cloning into mammalian expression vector Twist_CMV_VHHFc (Twist Bioscience). An immunoglobulin heavy chain signal peptide (MDWTWRILFLVAAATGAHS) was introduced at the N-termini of these proteins to facilitate secretion. Following transfection into Expi293 cells using the ExpiFectamine 293 Transfection Kit (both from Thermo Fisher Scientific), proteins hPTPRG-ECD-hFc, hPTPRG-N-hFc, hPTPRG-C-hFc, and mPTPRG-ECD-hFc were purified from supernatants using Protein A affinity chromatography and subsequently analyzed by SDS-PAGE and A280 absorbance, respectively. Additionally, human Nectin-2-ECD (amino acids 1–360) fused to hFc and hCD105-ECD (amino acids 1–586) fused to hFc were procured from Sino Biologics. Rabbit anti-human PTPRG pAbs were purchased from Thermo Fisher Scientific (cat. No. PA5-89705), while mouse anti-human Nectin-2 mAbs conjugated to APC (cat. no. 337411) and rabbit anti-human Nectin-2 pAbs (cat. no. 10005-RP03) were obtained from BioLegend and Sino Biologics, respectively. Mouse anti-human CD105 mAbs conjugated to APC (cat. no. 323208) and goat anti-human CD105 pAbs (cat. no. AF1097) were sourced from BioLegend and R&D Systems, respectively.

### Fab-phage biotinylation and capture

A detailed protocol for FBC using a naïve chimeric rabbit/human Fab library in phagemid pC3Csort [17] has been reported previously [14]. The same protocol and library were used here for three rounds of panning against 5 × 10^7^ Kasumi-1 cells. A first-round selection by conventional WCP was conducted to accumulate Kasumi-1 binders. A second-round selection by FBC was carried out in triplicates in parallel on 5 × 10^7^ Kasumi-1 cells or 5 × 10^7^ PBMCs followed by streptavidin-coated magnetic beads. For FBC, pooled phage was enzymatically biotinylated [10 μM Sortase A, 250 μM custom synthesized Gly_3_-biotin (LifeTein), and 10 mM CaCl_2_ for 15 min at 37°C] just before WCP.

### NGS library preparation and analysis

Phagemids were extracted from approximately 2 × 10^8^ of fresh *Escherichia coli* ER2738 panning outputs using a QIAprep Spin Miniprep Kit (Qiagen). Rabbit V_H_-encoding DNA was amplified by PCR using Phusion High-Fidelity DNA Polymerase (1× Buffer, 0.06 U/μL DNAP, 0.2 mM dNTPs, 0.4 μM primers, 150 ng template; cycling conditions: 96°C, 30 s followed by <24 cycles of 98°C, 30 s; 55°C, 30 s; 72°C, 30 s). The ~450-bp PCR products were subsequently subjected to gel purification using a MinElute PCR Purification Kit (Qiagen) and served as templates for a second PCR reaction, during which P5/P7 cluster-forming sites and adaptors were appended. The reaction conditions mirrored those of the initial PCR, except for using the antisense P5-universal primer and barcoded P7-Index-i sense primers. Distinct P7 indices were employed for each of the six parallel pannings in two triplicates. The template concentration for this second PCR was set at 0.2 ng/μL. The resulting ~500-bp PCR products were subjected to gel purification followed by Agencourt AMPure XP beads (Beckman Coulter) purification at a 1:1 volumetric ratio following the manufacturer’s protocol. The eluted products were collected in 30 μL water, pooled together, and subjected to analysis on a MiSeq sequencer (Illumina) utilizing MiSeq Reagent Kit v3 (150-cycle) in a single-end configuration.

### Phagemid retrieval and Fab expression

Around-the-horn PCR was carried out to recover phagemids using output DNA panning from Kasumi-1 as a template. Phosphorylated forward primers were designed to anneal to the immunoglobulin heavy-chain joining region (IGHJ). Reverse primers were designed to anneal to each HCDR3 (refer to Suppl. Table 6 for a complete list of all primer sequences). After PCR reactions [1× Q5 Reaction Buffer, 0.02 U/μL Q5 Hot Start High-Fidelity DNA Polymerase (New England Biolabs), 0.2 μM dNTPs, 0.25 μM phosphorylated IGHJ forward primer, 0.25 μM HCDR3-specific reverse primer, 20 pg template; cycling conditions: 98°C, 30 s followed by 35 cycles of (98°C, 10 s; 72°C, 3 min)], ~5 kb PCR products were purified using a MinElute PCR Purification Kit (Qiagen) and self-ligated with T4 DNA ligase (Roche). From each ligation reaction, 1 μL was utilized for the transformation of 10 μL Rosetta™ 2(DE3) Competent Cells (Novagen). Following bacterial recovery in SOC medium for 1 h at 37°C, each culture was plated on separate LB agar plates containing 100 μg/ml carbenicillin and 10 μg/ml tetracycline and incubated overnight at 37°C. Each colony was inoculated into 1 ml autoinduction media containing the same antibiotics and incubated overnight at 37°C to express Fabs.

### Expression profiling of hit Fabs using flow cytometry

Data were acquired using Accuri C6 flow cytometer (BD Biosciences) and analyzed with FlowJo 10.8.1 (BD Biosciences). Chimeric rabbit/human Fab genes on a pC3Csort vector of hits identified by FBC panning were sub-cloned into a modified pET11a expression vector by asymmetric *SfiI* cloning. The resulting pET11a constructs were transformed into Rosetta 2(DE3), and Fabs were expressed in 10 ml autoinduction medium [18] [SB medium supplemented with 2 mM MgSO_4_, 1 × 5052 solution (0.5% glycerol, 0.05% glucose, and 0.2% α-lactose), and 1× M solution (25 mM Na₂HPO₄, 25 mM KH₂PO₄, 50 mM NH₄Cl, and 5 mM Na₂SO₄)] supplemented with 50 μg/ml carbenicillin and 5 μg/ml chloramphenicol overnight at 37°C. After pelleting the bacteria and decanting the supernatant, Fabs expressed in the periplasm were extracted with 1 ml TSE buffer [200 mM Tris–HCl (pH 8.0), 500 mM sucrose, 1 mM EDTA]. After incubating for 30 min on ice, 50 μL Fabs in TSE buffer were taken and used for the staining of 1 × 10^5^ cells that were prepared for flow cytometry profiling. After Fab staining for 30 min on ice followed by washing with 150 μL flow cytometry buffer (PBS supplemented with 0.5% BSA, 2 mM EDTA, and 0.1% sodium azide), cells were then stained with 25 μL of a 100-fold dilution of PE-conjugated goat anti-human IgG, F(ab′)_2_ fragment-specific antibody (Jackson ImmunoResearch no. 109-116-097) in flow cytometry buffer. After 15-min incubation on ice, cells were washed twice with 150 μL flow cytometry buffer, and then resuspended in 100 μL flow cytometry buffer and analyzed. For PBMC analysis, the cells were gated and separated into three subpopulations based on FSC/SSC parameters: Sub 1 (lymphocytes), Sub 2 (monocytes), and Sub 3 (residual granulocytes), as shown in Fig. 2.

### IgG1 expression

Human V_H_, V_κ_, and V_λ_ genes of Fab hits were PCR-amplified from pET11a and NotI/NarI-cloned into modified pCEP4 expression vectors encoding corresponding heavy and light chain constant domains [19]. For the expression of chimeric rabbit/human IgG1, 25 μg heavy chain encoding pCEP4 and 25 μg light chain encoding pCEP4 were transfected to 50 ml Expi293F cell culture using the ExpiFectamine 293 Transfection Kit. Six days after transfection, IgG1s were purified by affinity chromatography using a CaptureSelect CH1-XL affinity matrix.

### CRISPR-Cas9 library screening

Loss-of-expression/binding screens were conducted using FACS with two AML cell lines transduced with a human improved genome-wide knockout CRISPR library (Addgene, #67989), comprising 90 709 sgRNAs targeting 18 010 human genes, with ~5 sgRNAs per gene. HEL and U937 cells were initially transduced with lentivirus carrying Cas9:Blst genes (Addgene, #68343). Blasticidin S-resistant cells were subsequently used to select single colonies expressing functional Cas9, which served as host cells to generate the sgRNA library as described [20]. In brief, 50 million cells were transduced at an multiplicity of infection (MOI) of ~0.3 to ensure single entry of lentivirus. HEL cells were sorted by flow cytometry for BFP-positive cells 72 h post-infection, while U937 cells were selected for puromycin resistance for stable integration over 5–7 days. Subsequently, 20 million BFP-positive HEL cells or puromycin-resistant U937 cells were stained with the newly discovered antibodies in chimeric rabbit/human IgG1 format or Fab format (AF647 labeled), respectively, across different categories, followed by negative selection using FACS. Interestingly, HEL cells negatively sorted by antibodies in the BLUE category (e.g. 2282 and 484) failed to proliferate, whereas U937 cells grew after selection. Following two rounds of sorting, cells were subjected to genomic DNA extraction for amplification of enriched sgRNAs. The PCR products containing the sgRNA expression cassettes were subsequently custom sequenced (Azenta, Amplicon-EZ) using NGS on a MiSeq instrument, yielding ~50 000 reads per sample. MAGeCK analysis was performed at the University of Florida bioinformatics core to identify the top enriched sgRNAs in each sample. The top-ranked sgRNAs were cloned into an sgRNA expression lentiviral vector (Addgene, #67974) and transduced into U937 cells to assess the knockout of target genes and loss of binding of the antibodies. To mitigate potential off-target effects, corresponding sgRNAs from the lentiCRISPR v2 library (Addgene, #52961) [35], targeting the same genes were also cloned and transduced into U937 cells.

### ELISA

Each well of a 96-well Costar 3690 plate (Corning) was coated with 100 ng of goat anti-human IgG Fcγ pAbs (Jackson ImmunoResearch) in 30 μL coating buffer (0.1 M Na_2_CO_3_, 0.1 M NaHCO_3_, pH 9.6) for 1 h at 37°C. After blocking with 150 μL 3% (w/v) bovine serum albumin (BSA)/PBS for 1 h at 37°C, 100 ng of Fc fusion proteins in 50 μL 1% (w/v) BSA/PBS were captured by incubation for 1 h at 37°C. The wells were washed three times with 150 μL PBS. Next, 100 ng of Fab in 50 μL 1% (w/v) BSA/PBS was applied to each well. Following incubation for 2 h at 37°C and washing as before, 50 μL of a 1:1000 dilution of Peroxidase AffiniPure™ F(ab')₂ Fragment Goat Anti-Human IgG, F(ab')₂ fragment specific (Jackson ImmunoResearch, #109-036-097) in 1% (w/v) BSA/PBS was added and incubated for 1 h at 37°C. The wells were washed four times, and colorimetric detection was performed using 2,2′-Azino-bis(3-ethylbenzothiazoline-6-sulfonic acid) (Sigma-Aldrich) as substrate according to the manufacturer’s directions. The absorbance was measured at 405 nm using a SpectraMax M5 microplate reader (Molecular Devices) and SoftMax Pro software (Molecular Devices).

### Surface plasmon resonance analysis

SPR experiments were conducted using a Biacore ×100 instrument along with Biacore reagents and evaluation software (Cytiva) to determine the kinetic and thermodynamic parameters of purified Fab-antigen interactions. A mouse anti-human IgG CH2 mAb was immobilized on a CM5 sensor chip according to the manufacturer’s instructions provided in the Human Antibody Capture Kit (Cytiva). Antigen proteins, either the full EC or specific subregions, fused to a human Fcγ1 domain were captured on the chip at a density not exceeding 400 RU. An empty reference flow cell was included on each sensor chip to allow for real-time background subtraction. All binding assays were performed using 1× HBS-EP+ running buffer [10 mM HEPES, 150 mM NaCl, 3 mM EDTA, pH 7.4, and 0.05% (v/v) Surfactant P20] at a constant flow rate of 30 μL/min. Fabs were injected at five different concentrations, including a duplicate of the lowest concentration to assess reproducibility. The sensor surface was regenerated using 3 M MgCl₂, as supplied in the Human Antibody Capture Kit, with no detectable loss of binding capacity. Kinetic parameters—association rate constant (*k*_on_) and dissociation rate constant (*k*_off_)—were determined by fitting the data to a 1:1 Langmuir binding model. The equilibrium dissociation constant (*K*_d_) was calculated as the ratio *k*_on_ / *k*_off_.

### Flow cytometry analysis of primary AML cells

Data were acquired using BD FACSCanto™ II (BD Biosciences) or MACSQuant® Analyzer 10 (Miltenyi Biotec) and analyzed with FlowJo 10.8.1 (BD Biosciences). Primary leukemic cells were harvested and washed twice with flow cytometry buffer (PBS supplemented with 0.5% FCS, 2 mM EDTA, and 1.5 mM sodium azide). Surface staining was performed for 25 min at 4°C in the dark. Following staining, cells were washed twice with flow cytometry buffer. Live cells were identified by exclusion of 7-AAD–positive cells. Where indicated, samples were pre-incubated for 25 min at 4°C with Human TruStain FcX™ Fc Receptor Blocking Reagent (BioLegend, prior to antibody staining to prevent non-specific Fc receptor–mediated binding).

### CAR design and vector construction

The scFv V_H_ and V_L_ sequences derived from Fab hits were custom synthesized by GeneArt (Thermo Fisher Scientific) and subcloned into a *Sleeping Beauty* transposon vector [21] to yield a second-generation CAR containing an IgG-based spacer domain, a CD28 transmembrane, a 4-1BB co-stimulatory domain, and a CD3 ζ signaling domain. The vector also contains a truncated epidermal growth factor receptor (EGFR) as a transfection marker, separated via a T2A sequence.

### CAR-T cell generation

CAR-T cells were generated using non-viral gene delivery with the *Sleeping Beauty* transposon system, as previously described [21]. In short, PBMCs from healthy donors were isolated from lymphocyte reduction chambers (provided by the Department for Transfusion Medicine of the University Hospital Würzburg) using density centrifugation. CD4^+^ and CD8^+^ T cells were enriched by CD4^+^ and CD8^+^ MicroBeads (Miltenyi Biotech). T cells were activated using Dynabeads Human T-Activator CD3/CD28 Beads at a bead:cell ratio of 1:1 and cultivated in CTL medium [RPMI 1640 medium supplemented with 1% (v/v) penicillin/streptomycin, 1× GlutaMAX-I, and 0.1% (v/v) 2-mercaptoethanol (all reagents from Thermo Fisher Scientific)] in the presence of 50 U/ml recombinant human IL-2 (rhIL-2) (Miltenyi Biotec). On Day 2, cells were nucleofected with a transposase (SB100X) and the CAR cassette-containing *Sleeping Beauty* transposon vector using a 4D-Nucleofector (Lonza) according to manufacturer’s instructions [22]. CAR-T cells were sorted for tEGFR expression and expanded for 10 days using irradiated PBMCs and TM-LCL feeder cells [23, 24], and 30 ng/ml anti-human CD3 antibody (Miltenyi Biotec).

### CAR-T cytotoxicity assays with AML cell lines

To enable detection of the cells by flow cytometry and by bioluminescence imaging, all cell lines were transduced with a lentiviral vector encoding a firefly luciferase (ffluc)_green fluorescent protein (GFP) transgene. Luminescence-based cytotoxicity assays were performed by co-incubating CD8^+^ CAR-T cells and ffluc-GFP fusion protein-expressing tumor cells in triplicate at different E:T ratios. D-Luciferin (Biosynth) was added to a final concentration of 0.15 mg/ml to allow luminescence signal measurement. Specific lysis was calculated in relation to Mock control cells.

### CAR-T cytotoxicity assays with primary AML cells

Bone marrow mononuclear cells (BMMCs) isolated from BM samples of AML patients were thawed, treated with 10 ng/ml DNase (Sigma-Aldrich), and rested for 24 h at 37°C, 5% CO_2_ in RPMI 1640 (Thermo Fisher Scientific, Waltham, MA) supplemented with 10% (v/v) fetal calf serum (FCS), 1% (v/v) GlutaMAX (Thermo Fisher Scientific), 100 U/ml penicillin/streptomycin (Thermo Fisher Scientific), 10 ng/ml SCF (Applied Biological Materials), and 10 ng/ml FLT-3 L (Thermo Fisher Scientific). AML cells were then co-cultured with CAR-T cells or Mock control cells at an E:T ratio of 2:1 and 1:1 for 24 h in triplicates. For analysis via flow cytometry, triplicates were pooled, washed with flow cytometry buffer, stained for surface antigens, and resuspended in 100 μl flow cytometry buffer +7AAD. Target cells were gated for CD3^−^ CD33^+^, while duplicated and dead cells were excluded. Lysis was determined as [(NumberTarget only – NumberTarget remain)/NumberTarget only] × 100%. “NumberTarget only” is the cell number of target cells without co-culturing. “NumberTarget remain” is the remaining cell number of target cells following co-culturing with CAR-T cells or Mock T cells.

### Cytokine release measurement

To assess cytokine secretion, CAR-T cells were co-cultured with target cells at an E:T ratio of 4:1 for 24 h. Then, the cell culture supernatant was harvested and ELISA MAX™ Kit (BioLegend) for IFNγ, and IL-2 was used according to manufacturer’s instructions.

## Results

### FBC-seq applied to Kasumi-1, an AML cell line

A naïve chimeric rabbit/human Fab library in pC3Csort, where a Sortase A recognition site (LPETGG) is fused to the C-terminus of the Fab light chain [14], was first selected with Kasumi-1, one of the AML cell lines classified as subtype M2 according to the French-American-British (FAB) classification, which assigns eight subtypes based on differentiation morphology [25]. The M2 subtype, myeloblastic with differentiation, is the most frequently found among AML patients. This first-round selection (the conventional WCP) is needed to roughly enrich the hit Fabs in the Fab-phage library prior to subjecting it to a second-round selection by FBC (Fab-phage biotinylation and capture). In this second-round selection, the biotinylated Fab-phage library was split and panned in parallel against Kasumi-1 cells and human healthy PBMCs for differential comparison (Fig. 1A). Based on previous findings that Fab clones can be identified by the heavy chain complementarity determining region 3 (HCDR3) [26], HCDR3-encoding DNA from selected phage was amplified and sequenced by NGS. Differential abundance analysis of Kasumi-1 cell binders against PBMC binders using HCDR3 as an identifier pinpointed a panel of Fab clones preferentially binding to Kasumi-1 cells (Fig. 1B). A cutoff (differential abundance >8 and *P*-value <1 × 10^−4^) yielded a collection of 192 HCDR3 identifiers predicted to bind antigens expressed on Kasumi-1 cells but absent on healthy PBMCs. To retrieve Fabs of interest from pooled phagemid outputs, an around-the-horn PCR approach was implemented [26] and 187 Fab-encoding phagemids were recovered. Flow cytometry screening of Fabs expressed from recovered phagemids revealed that 42 Fabs bound to Kasumi-1 cells.

**Figure 1:**
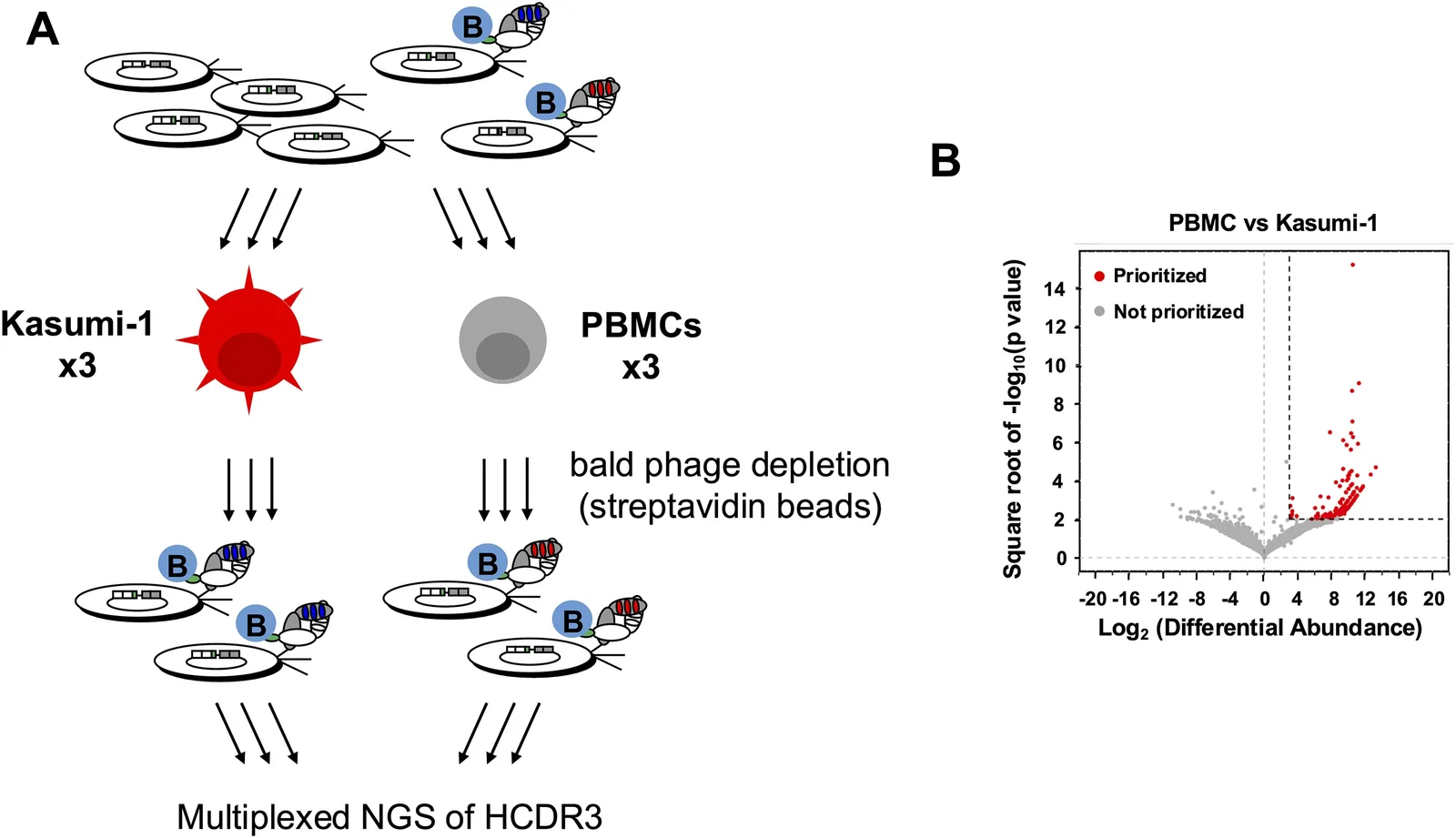
FBC-seq analysis between Kasumi-1 and PBMCs. (A) Schematic of FBC-seq. The workflow after the second-round selection is shown. Because the Fab was fused with a recognition tag for Sortase A, only phage displaying Fabs were biotinylated. This Fab-phage library was panned against Kasumi-1 cells or human healthy PBMCs followed by incubation with streptavidin magnetic beads, allowing the depletion of bald (i.e. Fab-less) phage. HCDR3 genes were amplified from each phage output and analyzed by NGS. (B) A volcano plot for visualizing the enrichment of HCDR3 identifiers in Kasumi-1 cell binders compared to PBMC binders. The *x*-axis represents the fold change of differential abundance, and the *y*-axis represents the statistical significance of this difference. The vertical dashed line indicates an eight-fold enrichment threshold (log_2_ differential abundance = 3). The horizontal dashed line indicates a *P* value threshold of 1 × 10^−4^ (square root of −log_10_[p value] = 2). Red data points, which exceed both thresholds, indicate HCDR3 identifiers that pass the cutoff filter.

### Kasumi-1 binders also bind to other AML subtypes

Further detailed analysis of the 42 Fab clones narrowed the candidate pool, excluding 14 clones: 5 carried incorrect HCDR3 identifiers, 5 displayed poor reproducibility, and 4 showed strong binding to PBMCs and T cells. We analyzed the remaining 28 Fab clones to determine whether they bind to additional 6 AML cell lines with different FAB classifications: TF-1 (M6), U937 (M4/M5), SKNO-1 (M2), MV4-11(M5), HEL (M6), and Molm13 (M5), in addition to Kasumi-1 (M2). We also included one chronic myeloid leukemia (CML) cell line, K562. The binding profile uncovered distinct patterns of reactivity (Fig. 2). Binding to PBMCs and hematopoietic stem cells (HSCs) was also tested, and no or only weak binding was detected, except for Fab clones #44 and #683, which bound HSCs strongly and were therefore excluded from the search for antibody and antigen candidates for AML therapy. Kasumi-1 binders fell into distinct categories, of which three are color-coded in Fig. 2 (BLUE, GREEN, and RED) as a guide for our subsequent target deconvolution efforts.

**Figure 2:**
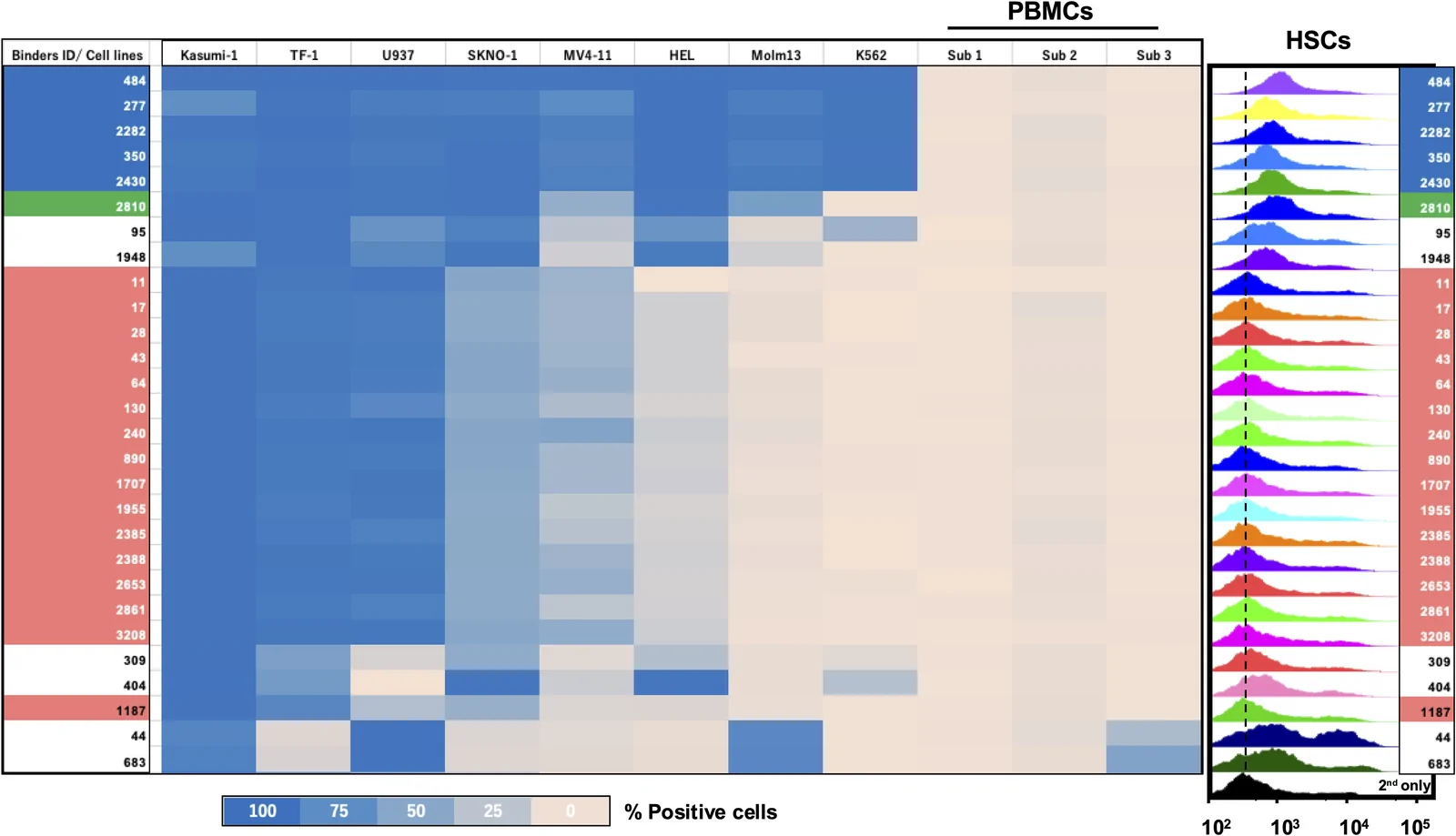
Binding profile of 28 Kasumi-1 binders. Left panel; a heatmap showing binding of Kasumi-1 binders to AML cell lines, CML cell line K562, and PBMC-derived subpopulations: lymphocytes (Sub 1), monocytes (Sub 2), and residual granulocytes (Sub 3). A threshold was set at the position where positive cells reached 5% in the control sample. “% Positive cells” was calculated by counting cells of which binding signals were greater than this threshold. The actual values are provided in the Suppl. Table 1. Right panel; flow cytometry histograms of binding to HSCs. Cells were stained using crude Fabs released from Rosetta™ 2(DE3). Crude periplasmic extracts containing Fabs were used directly for cell staining without normalization of Fab concentrations.

### Target deconvolution using a CRISPR-Cas9 knockout library

We have experienced that protein-based methods (e.g. IP/MS) have limitations for target deconvolution [15]. Considering the recent advancements in gene sequencing and editing technologies, methods focusing on analyzing the protein-encoding genes could be more dependable than directly interrogating the proteins. To pursue this concept, we opted to employ a CRISPR-Cas9 knockout library established through co-expression of Cas9 and a gRNA library. The key is that information on which genes are knocked out is tied to gRNA information. Cells subjected to the library become negative for a given antibody if the cognate antigen-encoding gene is knocked out. Such unstained cells are enriched by FACS to decode the antigen information from their genome (Fig. 3A). We chose the Human Improved Genome-wide Knockout CRISPR Library v1 designed by the Yusa laboratory because it has been widely and successfully used for screening in different cells, including AML cell lines [27]. For the target deconvolution campaigns, we used the HEL or the U937 rather than the Kasumi-1 cell line because of their faster proliferation.

**Figure 3:**
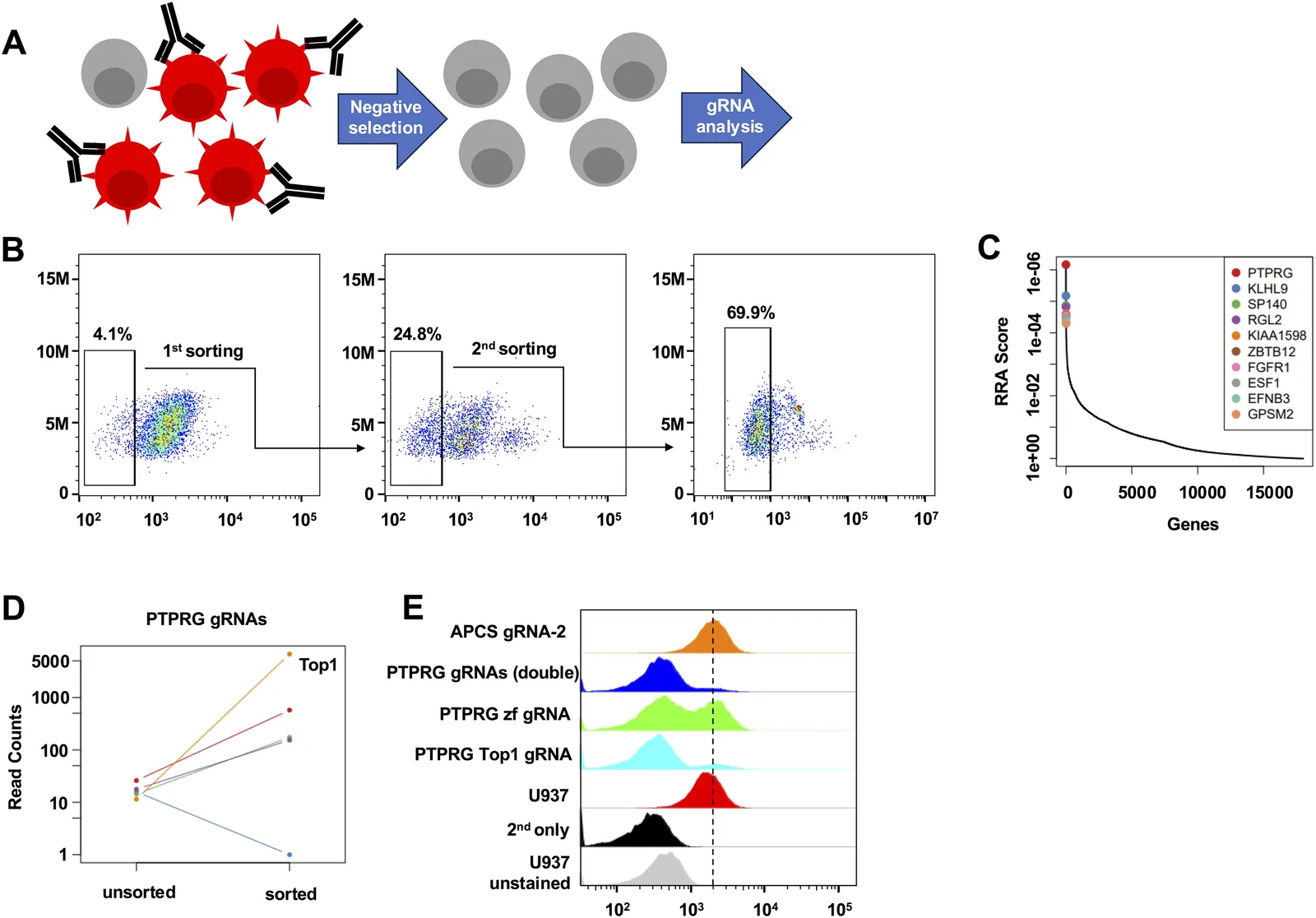
Target deconvolution of mAb_11 using CRISPR-Cas9 knockout library. (A) Schematic of target deconvolution using CRISPR-Cas9 knockout library. Candidate target genes were identified by sequencing gRNAs enriched in antibody-negative cells after sorting. In the HEL cell-based knockout library screen, IgG-format was used for negative selection, as illustrated in panel (A). For the other two screening categories using the U937 cell-based knockout library, the Fab format was used instead to avoid enrichment of FcγR-expressing cells. (B) Enriching process of HEL cell-based knockout library. Antigen-negative cells are enriched by sorting cells binding to mAb_11 in IgG format. The left-hand panel shows results before sorting, the middle panel shows results after the first round of sorting, and the right-hand panel shows results after the second round of sorting. (C) NGS results of gRNA genes extracted from enriched antigen-negative cells. MAGeCK analysis identified top gRNAs. The plot shows PTPRG ranked top 1 for mAb_11. (D) A plot showing that four out of five gRNAs targeting PTPRG were significantly enriched. (E) Flow cytometry histograms of the binding of Fab_11 to U937 cells. Orange; cells transduced with APCS gRNA-2 (negative control). Blue; cells transduced with two PTPRG gRNAs (Top1 and zf gRNAs). Green; cells transduced with PTPRG zf gRNA. Cyan; cells transduced with PTPRG Top1 gRNA. Red; parental U937 cells. The results of parental U937 cells treated with secondary antibody only (second only, black), and unstained U937 cells (gray) are also shown.

We started CRISPR-Cas9 knockout library screening based on HEL cells using mAb_11 in the RED category, the most abundant Fab clone according to FBC-seq analysis. When the original HEL library was stained with mAb_11 in IgG format, a small percentage (4.1%) of antigen-negative cells were detected and sorted (Fig. 3B). After two rounds of mAb_11 staining and sorting, the antigen-negative cells were sufficiently enriched (69.9%) to pursue genomic DNA extraction followed by PCR amplification and NGS of gRNA genes. Model-based Analysis of Genome-wide CRISPR-Cas9 Knockout (MAGeCK) [28] analysis revealed that several gRNA genes were enriched, the most prominent of which was PTPRG (Fig. 3C). Of the five PTPRG gRNAs in the library, four were significantly enriched after screening (Fig. 3D). To verify that mAb_11 does not bind to AML cells that do not express PTPRG, knockouts based on cell line U937 were established using the most enriched gRNA (Top1 gRNA) from the MAGeCK analysis, as well as an additional gRNA designed from a different CRISPR-Cas9 library (zf gRNA) from the Zhang laboratory [29]. U937 cells in which the PTPRG gene was knocked out by Top1 gRNA and zf gRNA were not or only partially, respectively, stained with mAb_11 (Fig. 3E, cyan and green, respectively). U937 cells double-knocked out with Top1 and zf gRNAs were also not stained with mAb_11 (Fig. 3E, blue). These findings reveal PTPRG as the antigen of mAb_11 and show that it is possible to identify the antigen by employing FACS to isolate unstained antigen-negative cells following CRISPR-Cas9 knockout library transfection.

Next, we validated PTPRG as the antigen of mAb_11 at the protein level. First, a recombinant protein encompassing human PTPRG-N structured domains (hPTPRG-N) as well as rabbit polyclonal antibodies (pAbs) against human PTPRG competed with the binding of mAb_11 to U937 cells (Fig. 4A). Second, direct binding of mAb_11 to hPTPRG-N, human PTPRG extracellular domain (hPTPRG-ECD), and mouse PTPRG extracellular domain (mPTPRG-ECD) was detected by surface plasmon resonance (SPR) (Fig. 4B). Notably, the Fab format of mAb_11 bound both species orthologs with single-digit nanomolar affinity.

**Figure 4:**
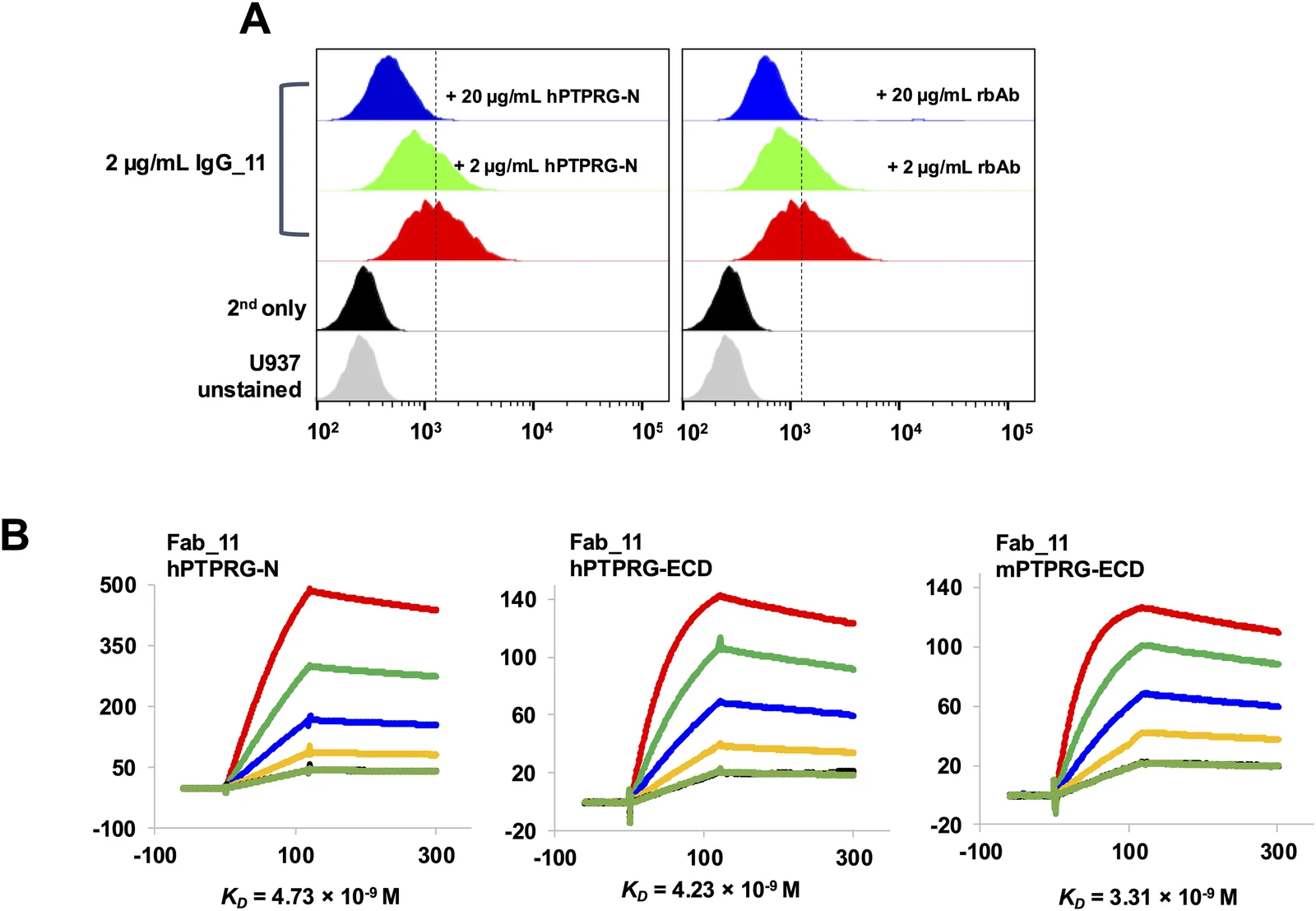
Characterization of mAb_11 using recombinant PTPRG protein. (A) Flow cytometry histograms of the binding of mAb_11 in IgG format in U937 cells in the presence of competing recombinant PTPRG protein (hPTPRG-N-hFc) (left panel) or rabbit anti-human PTPRG pAbs (rbAb; right panel), each at two different concentrations. Blue; cells treated with 2 μg/ml IgG_11 and 20 μg/ml of hPTPRG-N or rbAb. Green; cells treated with 2 μg/ml IgG_11 and 2 μg/ml of hPTPRG-N or rbAb. Red; cells treated with 2 μg/ml IgG_11 only. The results of U937 cells treated with secondary antibody only (second only, black), and U937 cells unstained (gray) are also shown. (B) SPR sensorgrams showing mAb_11 in Fab format bound the N-terminal structured domains of human PTPRG (hPTPRG-N-hFc) (K_D_ = 4.73 × 10^−9^ M) (left panel), the extracellular domain of human PTPRG (hPTPRG-ECD-hFc (K_D_ = 4.23 × 10^−9^ M) (middle panel), and the extracellular domain of mouse PTPRG (mPTPRG-ECD-hFc) (K_D_ = 3.31 × 10^−9^ M) (right panel).

### Target deconvolution of other clones in the RED category

Given the similarity in the binding profiles (Fig. 2) in the RED category, we hypothesized that they all bind to PTPRG. ELISA analysis showed that not only Fab_11 but also the other 12 clones of the RED category (Fab_17, Fab_28, Fab_64, Fab_130, Fab_240, Fab_1707, Fab_1955, Fab_2385, Fab_2388, Fab_2653, Fab_2861, and Fab_3208) strongly bound to hPTPRG-ECD, hPTPRG-N structured domains, and mPTPRG-ECD, but not to the C-terminal nonstructured region of human PTPRG (Fig. 5A). To elucidate whether all also shared an epitope with Fab_11, the binding of biotinylated Fab_11 to U937 cells was assessed using streptavidin-PE and in the absence or presence of unbiotinylated Fabs of the 12 clones. Flow cytometry analysis showed that 11 of the 12 Fabs competed with Fab_11, suggesting a shared or overlapping epitope. Only Fab_1955 revealed evidence for a non-overlapping epitope (Fig. 5B). Taken together, through ELISA, we successfully unveiled the targets of 12 Kasumi-1 binders in a swift manner, suggesting that similarities of binding profiles (Fig. 2) can accelerate target deconvolution.

**Figure 5:**
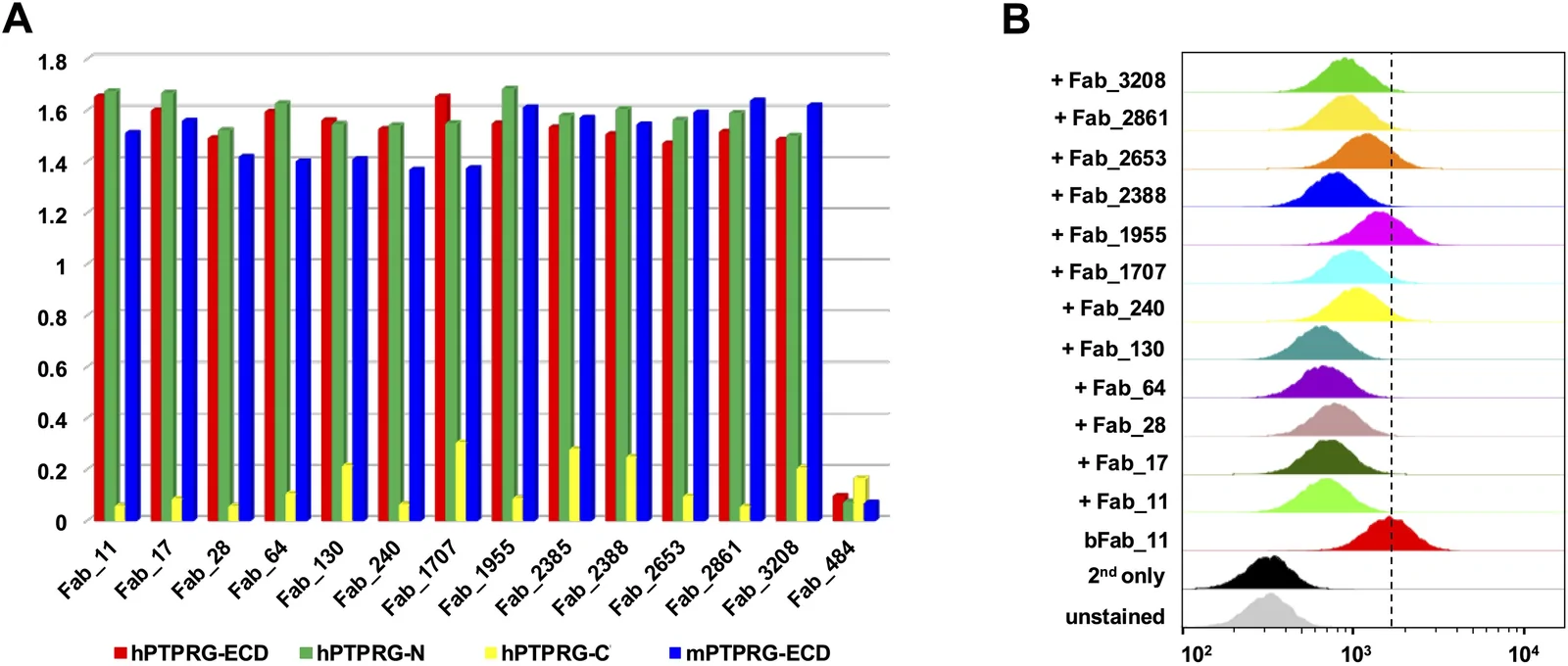
mAbs in RED category bound to PTPRG. (A) ELISA analysis to confirm binding to PTPRG, with hPTPRG-ECD-hFc, hPTPRG-N-hFc, hPTPRG-C-hFc, and mPTPRG-ECD-hFc used as antigens. The antibody clones categorized as RED (Fab_11, Fab_17, Fab_28, Fab_64, Fab_130, Fab_240, Fab_1707, Fab_1955, Fab_2385, Fab_2388, Fab_2653, Fab_2861, and Fab_3208) were tested. Fab_484 of the BLUE category was included as a negative control. (B) Epitope binning studies by flow cytometry. Binding of biotinylated Fab_11 to U937 cells was measured by streptavidin-PE, in competition with unbiotinylated Fab_3208, Fab_2361, Fab_2635, Fab_2388, Fab_1955, Fab_1707, Fab_240, Fab_130, Fab_64, Fab_28, Fab_17, and Fab_11. Binding of biotinylated Fab_11 to U937 cells with no unbiotinylated competitor Fab (bFab_11) was also measured as a positive control. The results of U937 cells treated with secondary antibody only (second only, black), and unstained U937 cells (gray) are also shown.

### Clone #1187 also binds to PTPRG

Outside of the RED category, we next took a closer look at clone #1187, which has a binding profile that is different from other Kasumi-1 binders (Fig. 2). Surprisingly, mAb_1187 did not bind to the enriched CRISPR-Cas9 knockout library after two rounds of negative selections with mAb_11, suggesting mAb_1187 may recognize the same antigen as mAb_11, PTPRG (Fig. 6A). Flow cytometry analysis using biotinylated IgG_11 or biotinylated IgG_1187 showed that they have a shared or overlapping epitope (Fig. 6B). The binding of mAb-1187 to U937 cells was competed by hPTPRG-N and by rabbit anti-hPTPRG pAbs (Fig. 6C). Binding to hPTPRG was confirmed by SPR (Fig. 6D), but the affinity of mAb_1187 (K_D_ = 1.90 × 10^−7^ M) was two orders of magnitude lower than that of mAb_11 (K_D_ = 4.23 × 10^−9^ M) (Fig. 4B). This may be the reason why mAb_1187 was not categorized as RED.

**Figure 6:**
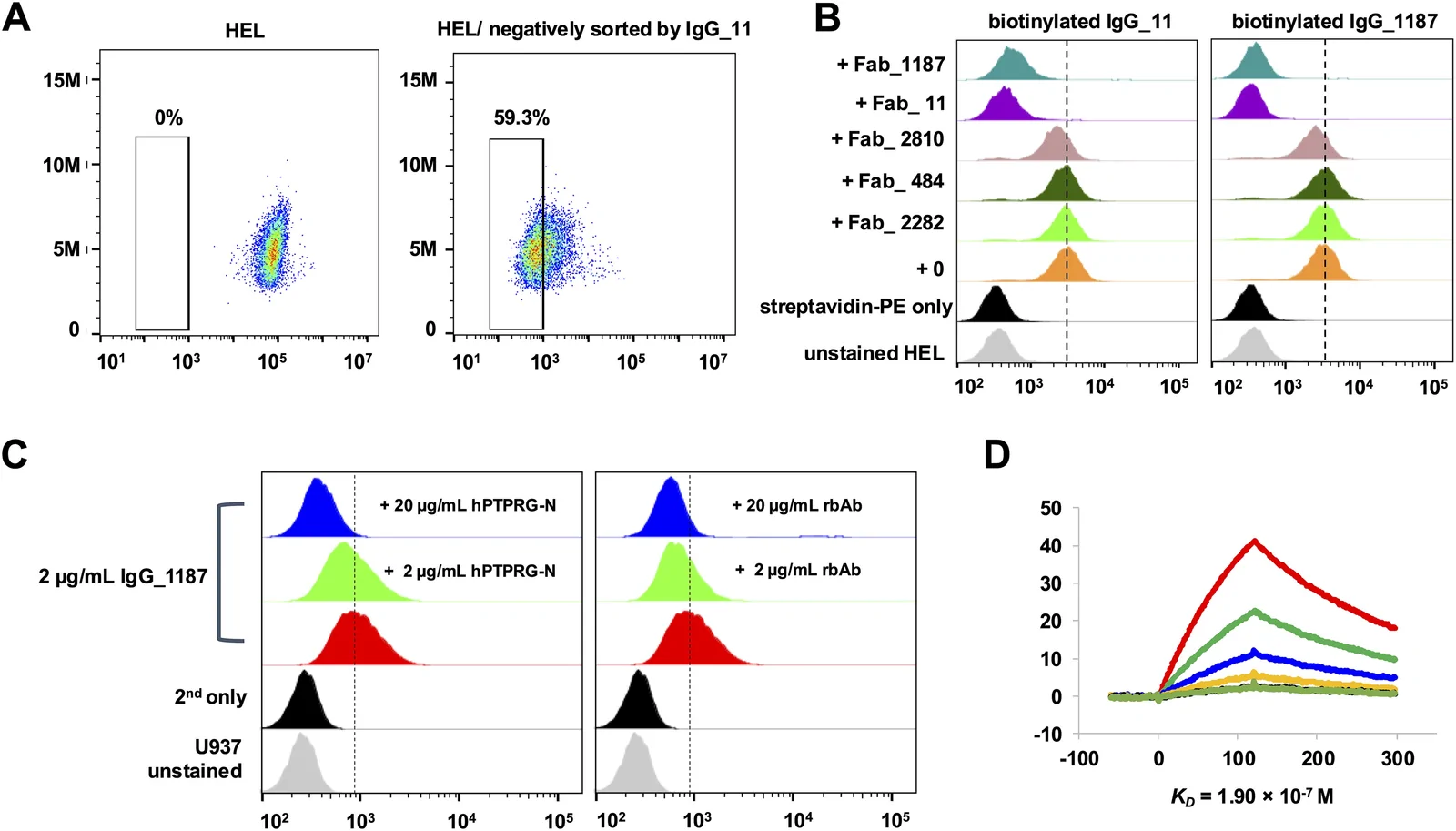
Target deconvolution of mAb_1187. (A) HEL parental cells (left panel) or HEL cells negatively sorted by IgG_11 (right panel) stained by biotinylated IgG_1187. (B) Epitope binning studies by flow cytometry. The binding of biotinylated Fab_11 (bIgG_11, left panel) and biotinylated Fab_1187 (bIgG_1187, right panel) to HEL cells was measured by streptavidin-PE in competition with unbiotinylated Fab_1187, Fab_11, Fab_2810, Fab_484, and Fab_2282. Binding of biotinylated IgG_11 or IgG_1187 to HEL cells with no unbiotinylated competitor Fab (+ 0) was also measured as a positive control. The results of HEL cells treated with streptavidin-PE only (black), and unstained HEL cells (gray) are also shown. (C) Flow cytometry histograms of the binding of mAb_1187 in U937 cells treated with recombinant PTPRG protein (hPTPRG-N-hFc) (left panel) or treated with rabbit pAbs targeting human PTPRG (rbAb) (right panel), each at two different concentrations. Blue; cells treated with 2 μg/ml mAb_1187 and 20 μg/ml of hPTPRG-N-hFc or rbAb. Green; cells treated with 2 μg/ml mAb_1187 and 2 μg/ml of hPTPRG-N-hFc or rbAb. Red; cells treated with 2 μg/ml IgG_1187 only. The results of U937 cells treated with secondary antibody only (second only, black), and U937 cells unstained (gray) are also shown. (D) SPR sensorgram showing the affinity of Fab_1187 to hPTPRG-ECD-hFc (K_D_ = 1.90 × 10^−7^ M).

### Target deconvolution of clones in the BLUE category

Applying the same approach used to identify PTPRG as the antigen of the RED category, we next attempted target deconvolution for the BLUE category, using the U937 cell line. Before or after one round of CRISPR-Cas9 library screening by Fab_2282 staining, the sorted library was predominately antigen-positive (Fig. 7A, red and green, respectively). As observed in the screening for the RED category, antigen-negative cells dominated after the second round (Fig. 7A, blue). NGS and MAGeCK analysis identified PVRL2 (a.k.a. NECTIN2) as the antigen candidate (Fig. 7B). All five gRNAs that target the NECTIN2 gene were highly enriched in the sorted compared to the unsorted library (Fig. 7C). Fab_2282 did not bind to U937 cells that were knocked out with the most enriched gRNA (Top1 gRNA), a NECTIN2 gRNA from a different CRISPR-Cas9 library (zf gRNA) [29], and a combination of both gRNAs (Fig. 7D). Binding of Fab_2282 to human Nectin-2 was confirmed by SPR (K_D_ = 5.97 × 10^−7^ M) (Fig. 7E).

**Figure 7:**
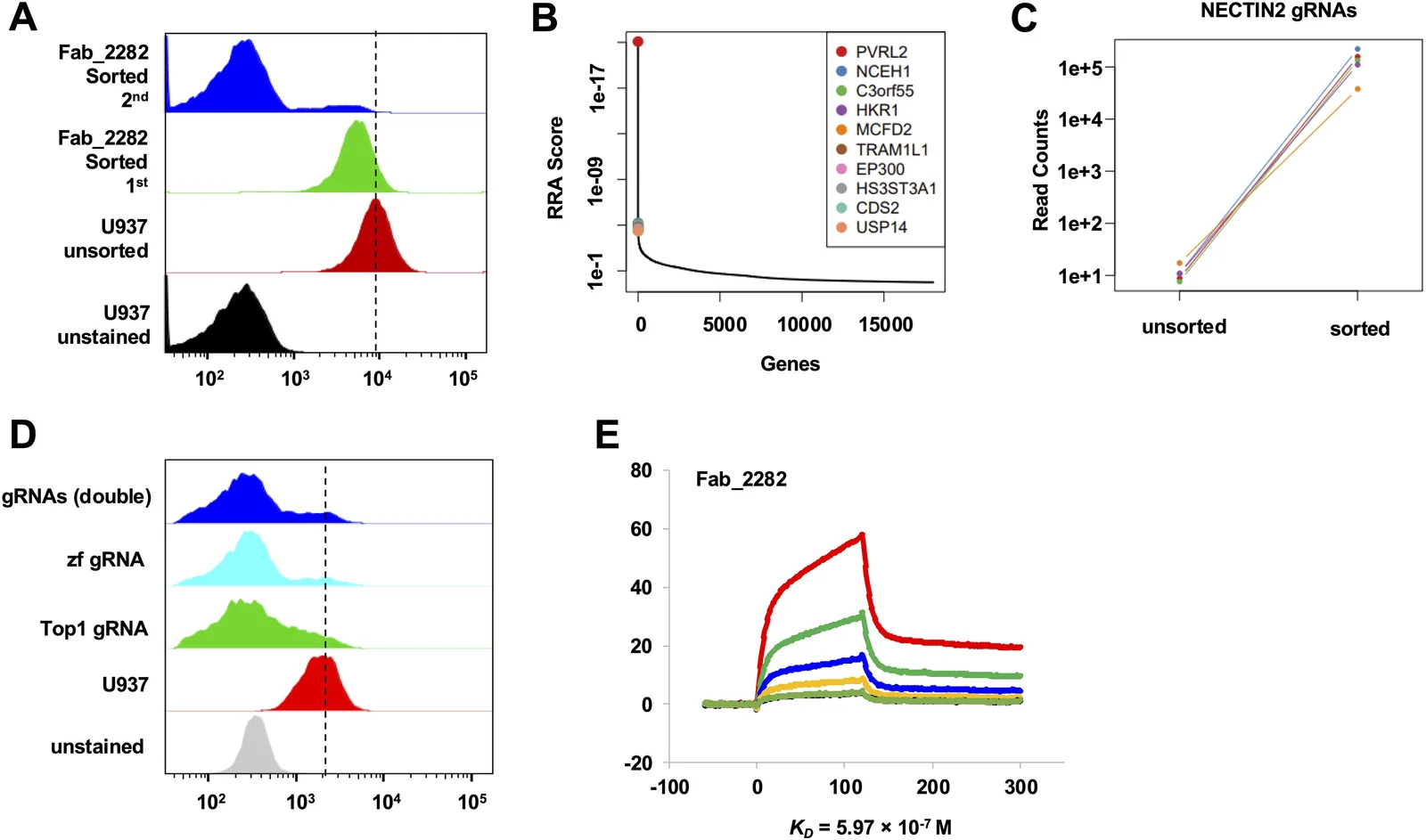
Target deconvolution of mAb_2282. (A) Binding studies of U937 cells transduced with CRISPR-Cas9 gRNA library and sorted by Fab_2282 to enrich antigen-negative cells. Flow cytometry histograms show the binding after two rounds of sorting (Fab_2282 sorted second, blue), after one round of sorting (Fab_2282 sorted first, green), unsorted U937 cells (U937 unsorted, red), and unstained U937 cells (U937 unstained, black). (B) NGS results of gRNA genes extracted from enriched antigen-negative cells. MAGeCK analysis identified PVRL2 (a.k.a. NECTIN2) ranked top 1 for clone 2282. (C) Plots showing that all five gRNAs targeting NECTIN2 were significantly enriched. (D) Flow cytometry histograms of the binding of Fab_2282 to U937 cells. Blue; cells transduced with two NECTIN2 gRNAs (Top1 and zf gRNAs). Cyan; cells transduced with NECTIN2 zf gRNA. Green; cells transduced with NECTIN2 Top1 gRNA. Red; parental U937 cells. Gray; unstained U937. (E) SPR sensorgrams showing the affinity of Fab_2282 to human Nectin-2 (K_D_ = 5.97 × 10^−7^ M).

Next, we investigated whether other clones categorized as BLUE (Fig. 2) bound to Nectin-2. Fab_484, Fab_277, and Fab_2430 failed to bind NECTIN2-knockout U937 cells, indicating that they most likely recognize Nectin-2 (Fig. 8A). We again used competitive flow cytometry analysis and all tested BLUE clones (Fab_484, Fab_277, and Fab_2430), but not a control GREEN clone (Fab_2810), competed with the binding of fluorophore-labeled Fab_2282 to U937 cells, indicating their binding to Nectin-2 with shared or overlapped epitopes (Fig. 8B). Furthermore, the binding of Fab_2282, Fab_484, and Fab_277 to U937 cells was competed by a human Nectin-2-human Fc (hNectin-2-hFc) fusion protein and by rabbit anti-human Nectin-2 pAbs (Fig. 8C–E). Exceeding Fab_2282 10-to-50-fold, as measured by SPR, double-digit nanomolar affinities of Fab_484 (K_D_ = 11.0 × 10^−9^ M), Fab_277 (K_D_ = 27.1 × 10^−9^ M), and Fab_2430 (K_D_ = 48.4 × 10^−9^ M) confirmed their binding to human Nectin-2 (Suppl. Fig. 1).

**Figure 8:**
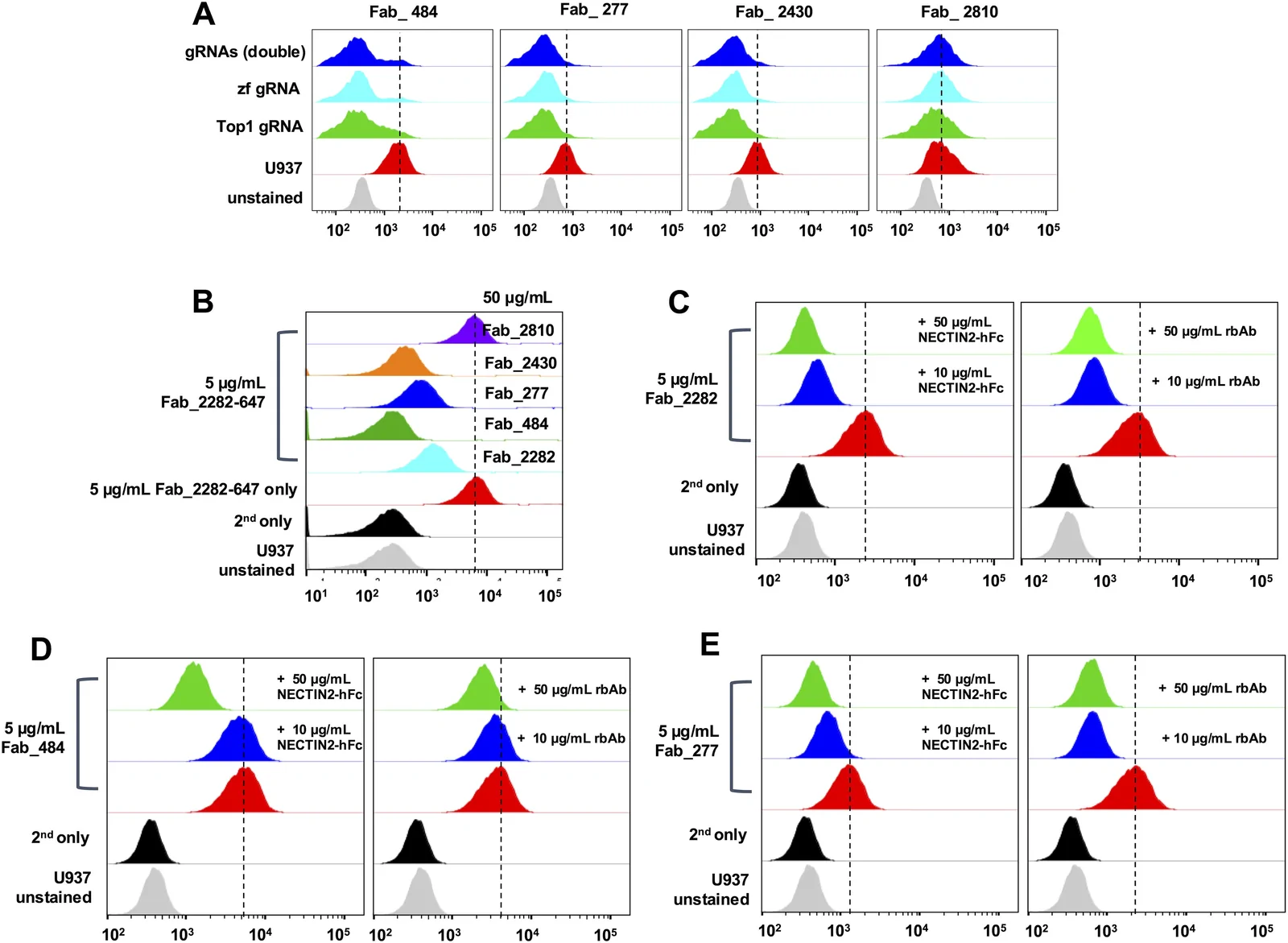
mAbs in BLUE category bound to Nectin-2. (A) Flow cytometry histograms of the binding of Fabs in the BLUE category (Fab_484, Fab_277, Fab_2430) to U937 cells. Binding of Fab in a different category (Fab_2810) was also measured as a control. Blue; cells transduced with two NECTIN2 gRNAs (Top1 and zf gRNAs). Cyan; cells transduced with NECTIN2 zf gRNA. Green; cells transduced with NECTIN2 Top1 gRNA. Red; parental U937 cells. Gray; unstained U937. (B) Epitope binning studies by flow cytometry. Binding of Alexa 647 labeled Fab_2282 to U937 cells in competition with unlabeled Fab_2430, Fab_277, Fab_484, and Fab_2282 itself. Binding of Alexa 647 labeled Fab_2282 with unlabeled Fab in a different category (Fab_2810) was also measured as a control. Binding of Alexa 647 labeled Fab_2282 with no unlabeled competitor (Fab 2282-647) is shown as a positive control. (C–E) Flow cytometry histograms of the binding of Fab_2282 (C), Fab_484 (D), and Fab_277 (E) to U937 cells, treated with hNectin-2-hFc [left panel of each of (C–E)] or treated with rabbit anti-human Nectin-2 pAbs [right panel or each of (C–E); rbAb], each at two different concentrations. Green; cells treated with 50 μg/ml of hNectin-2-hFc or rbAb. Blue; cells treated with 10 μg/ml hNectin-2-hFc or rbAb. Red; cells treated with 5 μg/ml each Fab in BLUE category only. Through (B–E), the results of U937 cells treated with secondary antibody only (second only, black), and unstained U937 cells (gray) are shown as negative controls.

### Target deconvolution of clone #2810 in the GREEN category

Finally, we pursued the identification of the antigen of clone 2810 categorized as GREEN. As before, following one round of CRISPR-Cas9 library screening using the U937 cell line and Fab_2810 staining, the sorted library exhibited predominantly antigen-positive cells (Fig. 9A, green), while the second round provided a sufficient number of antigen-negative cells (Fig. 9A, blue). NGS and MAGeCK analysis identified Endoglin (a.k.a. CD105) as the antigen candidate (Fig. 9B). Notably, all five gRNAs targeting the CD105 gene displayed a significant enrichment (Fig. 9C). Fab_2810 did not bind to U937 cells that were knocked out with the most enriched gRNA (Top1 gRNA) (Fig. 9D). However, Fab_2810 retained binding to U937 cells transduced with another gRNA (zf gRNA) from a different CRISPR-Cas9 library that was also predicted to target CD105, likely due to unsuccessful knockout by this gRNA. This interpretation was supported by staining with a commercial anti-CD105 mAb (Suppl. Fig. 2). Binding to a human CD105-human Fc (hCD105-hFc) fusion protein was confirmed by SPR (Fig. 9E). Binding of mAb_2810 to U937 cells was competed by hCD105-hFc and by goat anti-human CD105 pAbs (Fig. 9F).

**Figure 9:**
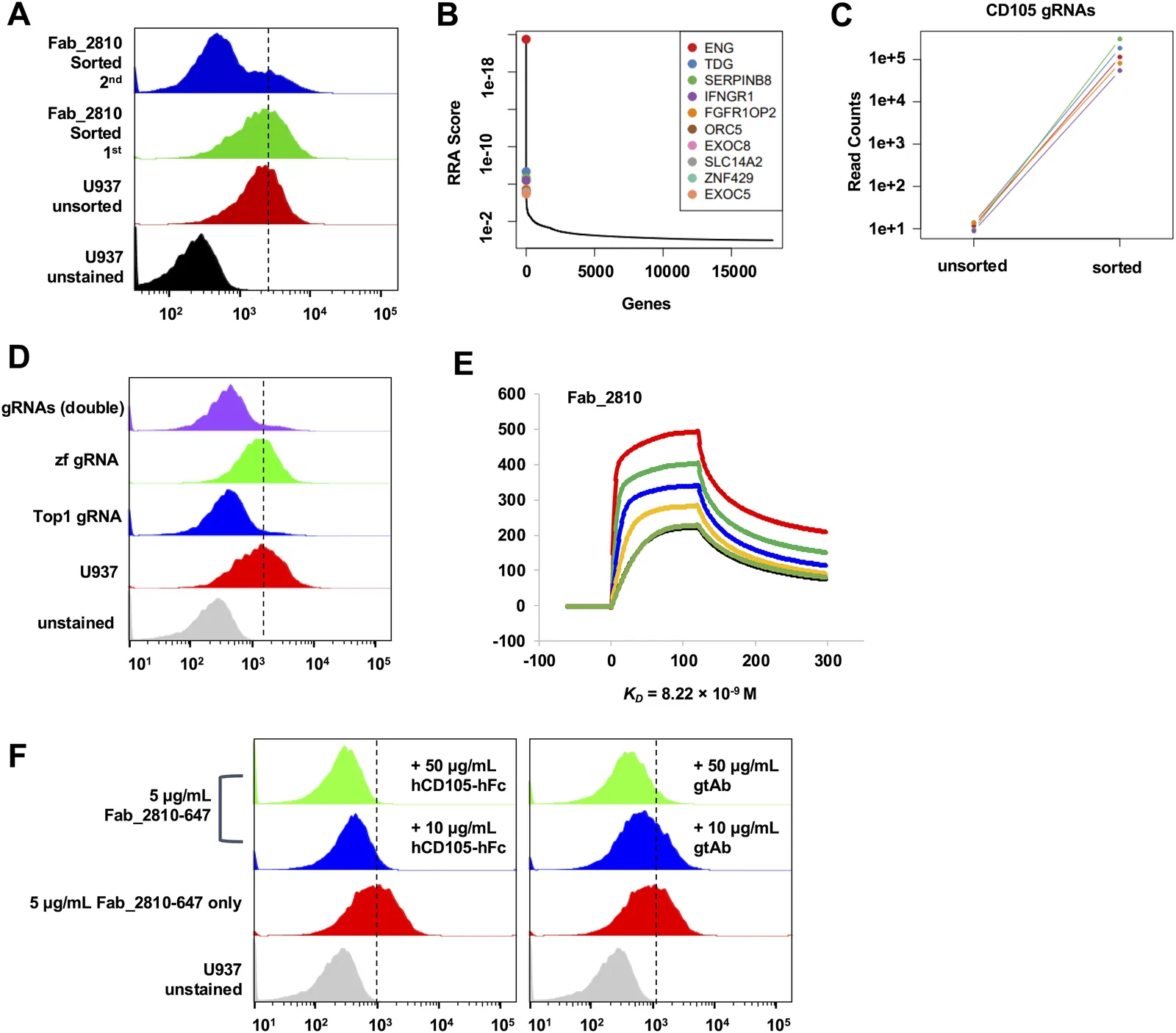
Target deconvolution of mAb_2810. (A) Binding studies of U937 cells transduced with CRISPR-Cas9 gRNA library and sorted by Fab_2810 to enrich antigen-negative cells. Flow cytometry histograms show the binding after two rounds of sorting (Fab_2282 sorted second, blue), after one round of sorting (Fab_2282 sorted first, green), unsorted U937 cells (U937 unsorted, red), and unstained U937 cells (U937 unstained, black). (B) NGS results of gRNA genes extracted from enriched antigen-negative cells. MAGeCK analysis identified Endoglin (ENG; a.k.a. CD105) ranked top 1 for clone 2810. (C) Plots showing that all five gRNAs targeting CD105 had been significantly enriched. (D) Flow cytometry histograms of the binding of Fab_2810 to U937 cells. Purple; cells transduced with two CD105 gRNAs (Top1 and zf gRNAs). Green; cells transduced with CD105 zf gRNA. Blue; cells transduced with CD105 Top1 gRNA. Red; parental U937 cells. Gray; unstained U937. (E) SPR sensorgrams showing the affinity of Fab_2810 to hCD105-hFc (K_D_ = 8.22 × 10^−9^ M). (F) Flow cytometry histograms of the binding of Fab_2810, treated with hCD105-hFc (left panel) or treated with goat pAbs targeting human CD105 (gtAb) (right panel) in a concentration-dependent manner. Green; cells treated with 50 μg/ml of hCD105-hFc. Blue; cells treated with 10 μg/ml of CD105-hFc. Red; cells treated with 5 μg/ml Alexa 647 labeled Fab_2810 only. The result of unstained U937 cells (gray) is shown as a negative sample.

### Summary of newly identified mAbs

To support future applications of our antibodies for AML research, detection, and treatment, we present specificity (Fig. 10A) and affinity (Fig. 10B) data, as well as the complete V_H_ and V_L_ amino acid sequences (Fig. 10C).

**Figure 10:**
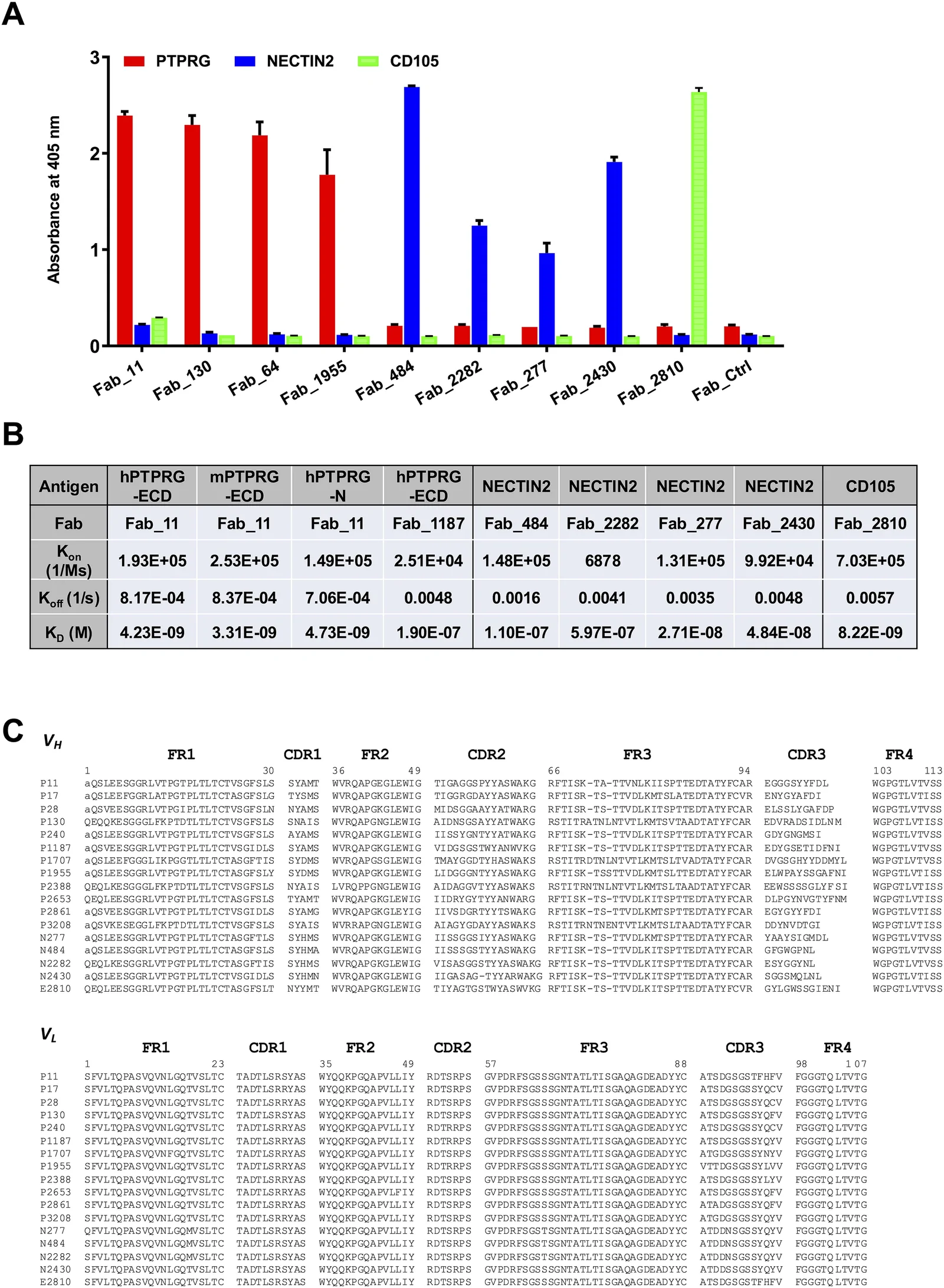
Summary of newly identified mAbs. (A) Binding of antibodies Fab_11, Fab_130, Fab_64, Fab_1955, Fab_484, Fab_2282, Fab_277, Fab_2430, Fab_2810, and control antibody (Fab_Ctrl) to each of PTPRG (red), Nectin-2 (blue), and CD105 (green) as measured by ELISA. (B) Table summarizing binding kinetics and affinities measured by SPR. (C) Amino acid sequence alignment of the rabbit variable domains (V_H_ and V_L_) of chimeric rabbit/human Fab clones is shown with framework regions (FRs) and complementarity determining regions (CDRs) using Kabat numbering. Clones starting with P bind PTPRG, clones starting with N bind Nectin-2, and clone starting with E binds CD105/endoglin.

### Verification of lead candidates on primary leukemic cells from AML patients

To demonstrate the potency of our approach in identifying clinically relevant immunotherapy targets and generating functional binders, we selected Fab lead candidates from each category (BLUE, GREEN, and RED) and reformatted them into full-length IgG1 molecules carrying LALAPG substitutions (L234A, L235A, P329G) to achieve efficient Fc silencing and thereby minimize background signals in flow cytometry analyses caused by Fcγ receptor interactions [30].

Primary leukemic cells were isolated from the BM of 12 AML patients, either at diagnosis or relapse, encompassing diverse mutational backgrounds, including complex cytogenetic abnormalities and FLT3 mutations, both associated with poor clinical outcomes (Suppl. Table 2). These cells were evaluated for binding by IgG1s 1955 (PTPRG), 484 and 2282 (Nectin-2), and 2810 (CD105). Notably, binding of all four antibodies was detected in both leukemic stem cell populations (CD38^−^CD34^+^) and bulk AML cells (CD38^+^CD34^+^), with 484 and 2810 showing the highest ΔMFI, highlighting their potential to target clinically relevant AML subsets (Fig. 11A and B). To further benchmark these new targets, we stained AML cells from an additional cohort of 10 AML patients (Suppl. Table 3) with commercially available flow cytometry antibodies against PTPRG, Nectin-2, and CD105 and compared them with antibodies targeting already established AML targets such as CD123, FLT3, and Siglec6. We observed binding to Nectin-2 and CD105 that was comparable to or, in some cases, exceeded that of the established benchmarks, underscoring the potential of these novel targets for therapeutic applications (Fig. 11C and Suppl. Table 3).

**Figure 11:**
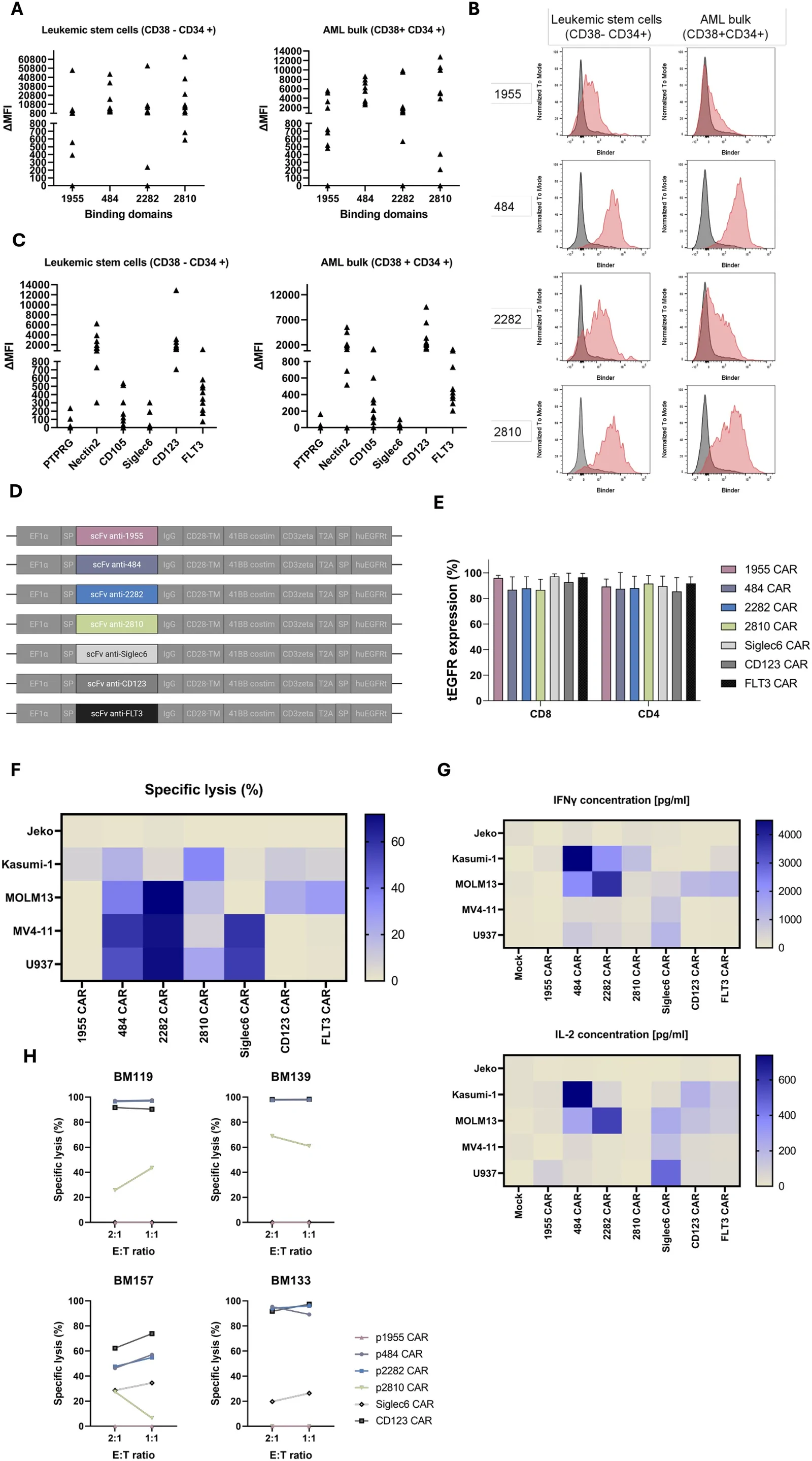
Binding to primary leukemic cells from AML patients and manufacturing and evaluation of second-generation CAR-T cells. (A) ΔMFI of primary AML cells (BM samples) stained with IgG1s (LALAPG) 1955 (anti-PTPRG), 484 (anti-Nectin-2), 2282 (anti-Nectin-2), and 2810 (anti-CD105) (*n* = 12). (B) Representative primary AML BM sample (BM143) stained with IgG1s (LALAPG) binding to 1955, 484, 2282, and 2810. Histograms show staining with IgG1 binders and secondary antibody (red) and the secondary antibody only control (gray). (C) ΔMFI of primary AML cells (BM samples) stained with commercially available antibodies against novel (PTPRG, Nectin-2, and CD105) and already established AML targets (Siglec6, CD123, and FLT3) (*n* = 10; PTPRG: *n* = 3). (D) Second-generation CAR constructs bearing 4-1BB as co-stimulatory domain were generated using potential new binders, alongside CARs recognizing established AML targets (Siglec6, CD123, and FLT3) for comparison. Truncated EGFR (tEGFR) was used as a transfection marker separated by a T2A sequence. (E) tEGFR expression of different CAR-T cells for CD8^+^ and CD4^+^ cells (*n* = 3; data is shown as mean ± SD). (F) Specific lysis (%) of target cells was determined after 6 h of co-culture with CD8^+^ CAR-T cells at an effector-to-target (E:T) ratio of 5:1. Specific lysis was calculated relative to Mock control T cells (*n* = 3). (G) IL-2 and IFNγ concentrations in supernatants were measured after 24 h of co-culture of CD4^+^ CAR T cells with target cells at an E:T ratio of 4:1 (*n* = 3). (H) CAR-T cell-mediated cytotoxicity was evaluated against primary AML cell (BM samples) using a flow cytometry-based assay. CD8^+^ CAR T cells were co-cultured with primary AML cells at two E:T ratios for 24 h, and the cell number of residual AML cells was quantified by flow cytometry. Specific lysis was calculated relative to Mock control cells.

To evaluate whether the newly generated binders could mediate effective tumor cell lysis, their sequences were incorporated as scFv binding domains into second-generation CAR constructs containing 4-1BB as the co-stimulatory domain (Fig. 11D). We then directly compared their anti-tumor efficacy with that of CAR-T cells targeting CD123, FLT3, and Siglec6. We observed comparable gene transfer efficiencies across all AML CAR constructs, as assessed by surface expression of the truncated epidermal growth factor receptor (tEGFR) transduction marker (Fig. 11E). CAR-T cells targeting Nectin-2 (484 and 2282) or CD105 (2810) effectively killed various AML cell lines in an antigen-specific manner (Fig. 11F and Suppl. Fig. 3). In contrast, CAR-T cells targeting PTPRG (1955) displayed cytotoxic activity only against Kasumi-1, probably due to a lack of target expression on the other tested AML cell lines (Fig. 11F and Suppl. Fig. 3). In addition, these CAR-T cells showed antigen-specific secretion of IL-2 and IFNγ upon stimulation with AML cell lines (Fig. 11G).

We next assessed the cytotoxic activity of the CAR-T cells against primary leukemic cells from AML patients (Suppl. Table 4). To do so, primary AML cells were co-cultured with either CAR-T cells or Mock control cells. The number of residual AML cells was quantified via flow cytometry after 24 h (Fig. 11H). PTPRG-targeting CAR-T cells failed to induce detectable cytotoxicity against AML cells derived from any of the tested BM samples, indicating that this antigen or antibody may not be suitable for CAR-T cell therapy in AML. In contrast, CD105-targeting CAR-T cells demonstrated moderate cytotoxicity in a subset of tested BM samples. CAR-T cells targeting Nectin-2 (484 and 2282) displayed efficient lysis of leukemic cells across all AML patients, comparable to CAR-T cells targeting CD123. However, this finding will need further validation in a larger patient cohort.

## Discussion

Phage display-based WCP, which utilizes cells as targets for antibody discovery, proves to be a potent tool in identifying candidate antigens for therapeutic purposes. While harnessing the advantage of target-agnostic drug discovery, we recognized the necessity of eliminating phage that non-specifically adsorb to the cell surface. For the FBC (Fab-phage biotinylation and capture) method [14], we created a Fab-phage library in which Fabs were recombinantly equipped with a Sortase A recognition site (LPETGG). This modification allowed us to selectively biotinylate Fab-displaying phage using Gly_3_-biotin, leading to the elimination of bald (Fab- and biotin-less) phage between the elution and re-amplification steps, hereby enhancing panning efficiency. Although this “Sortase A programmable” Fab-phage library facilitated the enrichment of positive clones, the initial implementation of FBC faced limitations due to the low-throughput nature of colony screening, typically confined to 10^2^–10^3^ clones. In contrast, selected libraries potentially contain 10^4^–10^7^ distinct clones. Consequently, Fab clones of interest with low abundance have the potential to be overlooked. To increase throughput, we decided to read the HCDR3 of selected Fab-phage pools using NGS. HCDR3 is generally the region of the highest diversity in antibodies and a crucial component of the paratope, giving it a function as an identifier [26]. Furthermore, to statistically narrow down the candidates for target deconvolution, we performed FBC not only on positive cells but also on negative cells in triplicates, followed by a subsequent statistical analysis for differential abundance. This improved WCP process, which we named FBC-seq [15], was employed here for the selection of Fab-phage that bind Kasumi-1 cells, with the overall goal of concerted antibody and antigen discovery for AML therapy.

Following FBC-seq, we started the screening of Kasumi-1 binders from 192 clones that showed statistical significance in differential abundance analysis. Although 187 phagemids (97%) were recovered by around-the-horn PCR using HCDR3-annealing primers, only 42 Fabs (22%) expressed from each recovered phagemid bound to Kasumi-1 cells. This highlights a limitation of the HCDR3-mediated recovery method; recovered clones potentially possess distinct sequences in the upstream regions of V_H_ or V_L_ and, as such, are different from NGS hits with the same HCDR3.

To refine the candidate pool prior to broader profiling across AML subtypes, we conducted a detailed analysis of the 42 initial Kasumi-1 binders. Five clones were excluded due to discrepancies between their retrieved HCDR3 sequences and the original identifiers, indicating possible PCR errors in phagemid retrieval. Another five binders showed limited reproducibility in Kasumi-1 staining, likely reflecting Fab instability, and were thus considered unsuitable for therapeutic development. Additionally, four clones exhibited strong binding to healthy PBMCs and T cells, raising concerns about off-target effects; these were also excluded from further consideration. As a result of this selection process, a total of 28 Fab clones were retained for further analysis.

Generating binding profiles for various cell lines proves highly valuable in predicting whether antibodies share a common antigen. In our previous study [15], we used binding profiles to identify four categories, and antibodies within the same category were found to bind to the same antigen. In the current study, we conducted target deconvolution for 3 categories across the remaining 28 Kasumi-1 binders, and, with only 1 exception, antibodies within the same category shared a common antigen. Clone #1187 was not assigned to the RED category, but shared PTPRG as the RED antigen, possibly due to its low affinity resulting in a weaker binding profile. Regarding Kasumi-1 binders shown as WHITE in Fig. 2, #44 and #683 were excluded from the inquiry due to robust binding to HSCs, an unfavorable feature for AML therapy. Other Kasumi-1 binders of the WHITE category were deprioritized due to a more restricted expression pattern among AML cell lines (Fig. 2). It is possible that some widely recognized AML targets, including CD123, which is expressed on HSCs, may have dropped out at this stage of the workflow.

For target deconvolution, we utilized a CRISPR-Cas9 knockout library. CRISPR-Cas9 libraries have proven to be potent tools for unraveling essential genes accountable for cancer severity [31], virus infection [32], or chromatin accessibility [33]. Notably, CRISPR-Cas9 libraries were successfully used to deconvolute the targets of mAbs with unknown specificities [34]. Generating a gene knockout library for a given cell line is relatively straightforward through the co-expression of Cas9 and a predesigned gRNA library. After applying selection pressure to the resulting cell library, the analysis of gRNAs integrated into the genome enables the identification of genes associated with a particular phenotype. Here, we utilized antibody staining followed by negative cell sorting for selective pressure. NGS analysis of gRNAs enriched in negative cell pools successfully identified the cognate antigens. This approach requires the presence of gRNAs in the library that target the gene encoding the antigen. Thus, careful library selection is crucial [35]. The approach also delivers hits that indirectly knock out or knock down the cognate cell surface antigen. However, by focusing on membrane proteins among the hits, this did not emerge as a shortcoming. In fact, indirect hits, which were not pursued in the current study, provide potential for elucidating aspects of the biology of the antigen.

Our antibody-driven antigen discovery campaign in AML resulted in identifying PTPRG, Nectin-2, and CD105. PTPRG belongs to the Protein Tyrosine Phosphatase (PTP) family. The protein consists of 1426 amino acids, with roughly half constituting its extracellular domain (ECD), followed by a single transmembrane region and 2 intracellular phosphatase domains. The ECD is equipped with a carbonic anhydrase (CAH)-like domain and a fibronectin type III domain. Interestingly, PTPRG was also identified as a hit in our previous multiple myeloma campaign, which used a multi-omic target deconvolution strategy [15], suggesting broader expression in hematologic malignancies. Notably, only one of the 16 anti-PTPRG Fab in the current study were selected in the previous study even though the same Fab-phage library was used for WCP. To our knowledge, there are no preclinical or clinical studies targeting PTPRG in AML, suggesting that our findings may open a new direction for the development of antibody-based therapies. Nectin-2, like PTPRG, is a glycoprotein with a single-pass transmembrane structure, and in its known functions acts as a calcium-independent cell adhesion molecule. The fully mature protein consists of 507 amino acids, featuring an immunoglobulin (Ig)-like V-type domain, an Ig-like C2-type 1 domain, an Ig-like C2-type 2 domain, a transmembrane domain, and a cytoplasmic domain. Recently, high expression of Nectin-2 has been reported as a marker of poor prognosis in AML [36]. In addition, engineered NK cells that exploit the Nectin-2–DNAM-1 axis have shown enhanced cytotoxicity against AML blasts [37]. These observations highlight Nectin-2 as a functionally relevant and therapeutically actionable surface antigen in AML, supporting the development of novel antibody-based strategies directly targeting pathogenic Nectin-2–expressing leukemic cells. CD105, commonly referred to as endoglin, is also a single-pass transmembrane glycoprotein involved in the regulation of angiogenesis. It is composed of 633 amino acids, encompassing an extracellular domain, a transmembrane domain, and a cytoplasmic domain. CD105-positive primary leukemic blasts have been reported to possess enhanced leukemogenic potential. Furthermore, treatment with an anti-CD105 antibody has been shown to inhibit AML development and progression, as confirmed in xenograft mouse models [38]. CD105 was also identified in another study that performed whole-cell panning using ovarian cancer cell lines, suggesting that CD105 may serve as a broadly applicable cancer target accessible to phage-displayed antibodies [39].

To evaluate the transcript-level expression of the identified targets, we used GEPIA3 [40] to compare TCGA-LAML tumor samples with a combined normal reference comprising paired peritumor samples from TCGA and corresponding normal samples from the Genotype-Tissue Expression (GTEx) project (Suppl. Fig. 4). Among the three identified targets, PTPRG and ENG (CD105) showed higher transcript expression in TCGA-LAML samples than in GTEx normal blood samples, whereas Nectin-2 was also highly expressed at the transcript level in normal blood. The relatively high expression of Nectin-2 in normal blood underscores the need for careful evaluation of potential on-target, off-tumor toxicity and comprehensive safety profiling before its therapeutic applicability can be established.

Nevertheless, a central feature of our screening strategy was that whole-cell phage display selections were performed separately against AML cells and healthy donor PBMCs, followed by statistical comparison of the antibody clone enrichment profiles between the two groups. Thus, the preferential binding of the selected antibodies to AML cells, despite the absence of AML-specific Nectin-2 overexpression at the bulk transcript level, may reflect differences between transcript abundance and accessible cell-surface antigen presentation. Such differences may arise from posttranscriptional regulation, variation in cell-surface protein density, differential epitope presentation, or the cellular heterogeneity inherent to bulk RNA-sequencing datasets.

Notably, CD33, a clinically validated and extensively investigated therapeutic target in AML, also showed substantial transcript expression in normal blood samples (Suppl. Fig. 4). This observation illustrates that AML-specific overexpression at the bulk RNA level is not an absolute prerequisite for the therapeutic targeting of a cell-surface antigen.

We next assessed the functional relevance of the three identified targets using CRISPR-Cas9 dependency data from the DepMap dataset [41, 42] (Suppl. Table 5 and Suppl. Fig. 5). None of the three genes showed evidence of a broad cell-intrinsic dependency across AML cell lines. However, the absence of such dependency does not preclude their use as therapeutic targets, because immune effector functions [including antibody-dependent cellular cytotoxicity (ADCC), antibody-dependent cellular phagocytosis (ADCP), and complement-dependent cytotoxicity (CDC)], bispecific immune-cell engagers, CAR-T cell therapies, ADCs, and radioimmunoconjugates (RICs) can eliminate antigen-positive cells regardless of whether the target itself is required for tumor-cell survival. Consistent with this concept, we demonstrated that CAR-T cells targeting Nectin-2 or CD105 lysed primary leukemic cells obtained from patients with AML (Fig. 11).

One question is why we did not identify Fabs against common AML targets such as CD33, CD123, or CD70. Aside from the flow cytometry filtering noted above, and as previously suggested, our selection appears biased toward antigens with larger ECDs [15]. Consistent with this, the deconvoluted targets in this study—PTPRG, Nectin-2, and CD105—possess substantially larger ectodomains than CD33, CD123, and CD70, which may have contributed to their preferential recovery during WCP.

Because the antibodies were selected against intact cells, some may recognize conformational epitopes that are influenced by glycosylation or other posttranslational modifications. Although target knockout confirmed the antigen dependence of antibody binding, we did not determine whether the recognized epitopes include posttranslationally modified residues. AML-associated alterations in glycosylation or epitope accessibility may therefore contribute to the observed preferential binding and should be investigated in future studies.

Although the repertoire of antibody-based cancer therapeutics is steadily expanding, there remains a significant bias in terms of their targets. An antigen-agnostic, “antibody-first” approach has the potential to overcome biases of the now more common “antigen-first” approach. In the current study, we profiled 28 binders and found 3 unexpected antigens. In the future, the application of long-read sequencing and single-cell sequencing of phage display and other antibody libraries could enable the identification of a larger pool of binders, facilitating the discovery of more antigens. When using CRISPR-Cas9 library screening, we performed cell sorting simply based on loss of binding. Alternatively, it may be possible to identify agonistic and antagonistic antibodies by screening for gain or loss of function.

Even though primary leukemic cells were not used during the initial target identification and binder generation, we were able to validate the lead candidates by staining primary cells derived from AML patients. Our results confirmed PTPRG, Nectin-2, and CD105 as candidates for targeted therapy for AML, due to their expression on both AML bulk cells and LSCs. Targeting LSCs is considered critical for the achievement of effective therapy, as these cells are thought to be responsible for the disease initiation, relapse, and treatment resistance [2]. Among the identified candidates, Nectin-2 showed particularly favorable expression characteristics, with expression on both newly diagnosed (*n* = 7) and relapsed (*n* = 4) AML patients, indicating that Nectin-2 expression can persist after prior therapy and supporting its potential as a stable and clinically relevant CAR-T cell target. Compared to Siglec6 and FLT3, Nectin-2 displayed higher expression on AML bulk cells and LSCs, and an expression level comparable to CD123. While CAR-T cell therapy targeting CD123 has been shown to be effective, hematopoietic toxicities have been observed, due to the target expression on HSCs, partly making HSC transplantation necessary to reconstitute normal hematopoiesis after adoptive cell therapy [43–45]. Importantly, in contrast to CD123, HSCs lacked detectable Nectin-2 expression, suggesting a potentially reduced risk of hematopoietic toxicity. These expression characteristics make Nectin-2 a promising target for CAR-T cell therapy in AML.

To further evaluate the therapeutic potential of the identified antibodies and antigens, we generated CAR-T cells directed against PTPRG, Nectin-2, and CD105 and tested their anti-tumor activity *in vitro*. Whereas CAR-T cells targeting Nectin-2 and CD105 revealed robust cytotoxicity, PTPRG-targeting CAR-T cells only showed limited anti-tumor efficacy against AML cell lines. Notably, in cytotoxicity assays with primary AML cells, Nectin-2-targeting CAR-T cells showed comparable anti-tumor activity to CAR-T cells targeting CD123. CD105-directed CAR-T cells also exhibited measurable cytotoxicity but were consistently less effective than CAR-T cells targeting Nectin-2 and CD123, suggesting more limited therapeutic utility.

Summarizing, of the three target candidates identified in our initial screening, only PTPRG failed to demonstrate significant anti-tumor activity in downstream evaluations. In contrast, our findings highlight CD105, and in particular, Nectin-2 as promising immunotherapeutic targets in AML. To enable clinical translation, further studies are needed, including validation in a larger and more diverse patient cohort, *in vivo* assessment in relevant AML models, and comprehensive toxicity and safety profiling.

## Supplementary Material

260813_Supplementary_tbag043

## Data Availability

Raw FASTQ data have been deposited in the NCBI Sequence Read Archive under BioProject accession number PRJNA1467206 and will be made publicly available upon publication. The R code used to analyze MiSeq FASTQ files is available at https://github.com/hedawils/FBCseq.

## Animal research statement

Not applicable. This study did not involve animal experiments.
