# Supplementary material for: Discovery of candidate antibodies and antigens for acute myeloid leukemia therapy by combining whole-cell phage display selection and CRISPR-Cas9 library screening
Source: Antib Ther. 2026 Sep 12;9(4):576–94. doi: 10.1093/abt/tbag043 (PMC13628138; doi:10.1093/abt/tbag043)
Supplement: Supplementary file 1 [file 260813_supplementary_tbag043.pdf]

Supplementary Tables

Suppl. Table 1 | Binding profile of 28 Kasumi-1 binders.

Clone/ Cell lines	Kasumi-1	TF-1	U937	SKNO-1	MV4-11	HEL	Molm13	K562	Sub 1	Sub 2	Sub 3
Fab_484	95.75	94.93	94.77	94.56	93.88	95.07	94.52	93.91	0.2	3.8	-0.63
Fab_277	72.75	94.43	87.77	87.36	75.28	95.07	86.72	93.61	0.1	1.8	0.25
Fab_2282	93.05	94.73	92.17	92.46	85.98	94.97	91.32	93.61	-0.7	5.2	-0.56
Fab_350	90.85	94.53	89.27	93.76	82.48	95.07	86.82	93.31	0.2	4.7	-1.23
Fab_2430	91.75	94.83	92.57	92.96	84.78	95.07	90.52	93.51	0.1	5.5	0.61
Fab_2810	95.05	94.33	92.27	94.56	44.08	94.57	58.72	-0.58	-0.1	3	0.35
Fab_95	95.95	94.23	67.97	85.16	25.48	66.97	7.92	39.31	-1.75	3.1	-0.73
Fab_1948	71.75	93.13	77.27	92.76	15.88	89.67	15.52	-0.65	-0.6	4.2	-0.3
Fab_11	96.25	91.33	92.87	50.66	39.68	-2.13	2.09	0.46	-1.44	-0.5	-1.09
Fab_17	95.75	89.43	87.47	47.36	37.78	15.77	4.68	-1.5	-0.7	5.1	-0.53
Fab_28	95.75	89.43	87.47	47.36	37.78	15.77	4.68	-1.5	-0.7	3.8	-0.73
Fab_43	96.15	91.43	89.77	50.56	39.08	15.57	-0.1	-0.15	-1.09	2.6	-0.66
Fab_64	96.05	91.13	89.17	50.66	42.48	15.27	4.19	-1.04	-0.7	3.8	-0.7
Fab_130	95.85	89.03	78.27	47.36	31.98	12.57	3.25	-1.07	-0.1	3	-0.83
Fab_240	95.95	91.93	92.67	50.66	52.68	17.47	5.42	-0.86	-1.02	3.9	-0.1
Fab_890	96.05	90.93	89.77	50.06	35.08	15.27	2.42	0.56	0.4	3.7	-0.42
Fab_1707	96.15	90.83	91.97	49.76	35.98	16.87	1.81	0.89	0	2	-1.21
Fab_1955	95.75	88.13	91.27	47.36	22.68	12.87	3.73	-0.83	-0.2	3.5	0.03
Fab_2385	96.05	90.23	84.87	48.16	27.08	13.37	2.01	-2	0	4.9	-0.58
Fab_2388	95.95	92.03	90.87	48.06	38.48	14.97	2.69	-2.05	-0.1	2.6	-0.25
Fab_2653	95.95	91.53	91.37	47.66	42.68	13.57	1.62	1.25	-1.45	3.3	-0.51
Fab_2861	96.05	90.03	84.97	47.76	24.18	14.47	1.21	0.43	0.8	2.8	-0.21
Fab_3208	96.25	92.03	92.67	49.96	45.78	17.07	1.06	-0.26	-0.5	0.8	0.67
Fab_309	95.25	57.43	12.97	46.66	6.58	29.17	3.66	6.71	0.3	3.5	-0.73
Fab_404	94.85	62.33	-2.03	93.96	18.38	94.57	3.96	28.31	-0.5	3.6	-0.65
Fab_1187	95.35	79.43	29.27	42.06	10.28	10.67	3.14	-0.46	-0.3	4.3	-0.86
Fab_44	80.45	8.83	94.87	10.26	6.58	3.78	78.22	-1.1	0.9	5.6	32.83
Fab_683	84.25	11.43	94.87	9.16	7.78	4.46	78.72	-0.92	-0.1	3.9	53.83

Suppl. Table 2 | AML patient characteristics (flow cytometry cohort 1)

BM Sample	Age	Sex	Disease Status	Molecular mutations	Cytogenetics	Blasts (%)	Secondary AML
AMLBM104	66	male	Relapse	CEBPA, TET2	complex karyotype	15-20%	No
AMLBM106	68	male	Primary diagnosis	ASXL1, CBL, GATA2, PHF6, SETBP1, SRSF2	normal karyotype	>30%	Yes
AMLBM117	66	male	Primary diagnosis	DNMT3A	complex karyotype	>90%	Yes
AMLBM121	60	male	Relapse	NRAS	complex karyotype	>90%	No
AMLBM123	66	male	Primary diagnosis	DNMT3A	NA	50%	Yes
AMLBM131	57	male	NA	BCOR, BCORL1, IDH2, NRAS, FLT3-ITD	NA	>90%	Yes
AMLBM135	68	female	Relapse	RAD21, RUNX1	complex karyotype	10-20%	Yes
AMLBM137	70	female	Primary diagnosis	KMT2A, IDH1	normal karyotype	20%	Yes
AMLBM139	67	male	Primary diagnosis	ASXL1, IDH2, RUNX1, SRSF2, STAG2, NRAS, PHF6 , PRPF8 , ZRSR2	complex karyotype	10-20%	Yes
AMLBM141	43	male	Relapse	NPM1, DNMT3A	NA	20%	No
AMLBM143	67	male	Primary diagnosis	BCOR, CHEK2, RUNX1, SRSF2, TET2	complex aberrant	>90%	No
AMLBM157	50	female	Primary diagnosis	ASXL2, SMC3, FLT3-TKD	complex aberrant	50%	No

DNMT3A: DNA methyltransferase 3 alpha; IDH2: isocitrate dehydrogenase 2; RUNX1: Runt-related transcription factor 1; FLT3: Fms-related receptor tyrosine kinase 3; NPM1: nucleophosmin 1; ASXL1: additional sex combs like 1; BCOR: BCL6 corepressor; NRAS: neuroblastoma RAS viral oncogene; TET2: Tet methylcytosine dioxygenase 2; IDH1: isocitrate dehydrogenase 1; CBL: Cbl proto-oncogene; CEBPA: CCAAT enhancer binding protein alpha; PHF6: plant homeodomain (PHD) finger protein 6; FLT3-ITD: FLT3 internal tandem duplication; GATA2: GATA binding protein 2; SETBP1: SET binding protein 1; SRSF2: serine and arginine rich splicing factor 2; BCORL1: BCL6 corepressor-like 1; RAD21: RAD21 cohesin complex component; KMT2A: lysine methyltransferase 2A; PRPF8: pre-mRNA-processing-splicing factor 8; ZRSR2: zinc finger CCCH-type RNA splicing factor 2; CHEK2: checkpoint kinase 2; SMC3: structural maintenance of chromosomes 3;

Suppl. Table 3 | AML patient characteristics (flow cytometry cohort 2)

BM Sample	Age	Sex	Disease Status	Molecular mutations	Cytogenetics	Blasts (%)	Secondary AML
**AMLBM0043**	88	male	Primary diagnosis	KMT2A-PTD	NA	50%	No
**AMLBM0047**	52	female	Primary diagnosis	DNMT3A, NRAS, TET2	normal karyotype	>90%	No
**AMLBM0048**	67	male	Relapse	NA	NA	60-70 %	Yes
**AMLBM0049**	75	male	Primary diagnosis	NPM1	NA	70%	No
**AMLBM0052**	66	female	Primary diagnosis	ATRX, BCOR, BCORL1, IDH2, RUNX1, SRSF2	normal karyotype	80%	No
**AMLBM0053**	63	male	Primary diagnosis	NPM1, PTPN11, RAD21, ASXL1	normal karyotype	NA	Yes
**AMLBM0054**	61	male	Primary diagnosis	NA	normal karyotype	30%	No
**AMLBM0060**	57	male	Primary diagnosis	BCOR, BCORL1, DNMT3A, NRAS, U2AF1	del(16q22) / normal karyotype	>90%	Yes
**AMLBM0062**	55	male	Primary diagnosis	IDH2, NF1, SMC1A	NA	>90%	No
**AMLBM0064**	73	male	Primary diagnosis	KRAS, NRAS, SRSF2, TET2	monosomy 7	20%	Yes

DNMT3A: DNA methyltransferase 3 alpha; IDH2: isocitrate dehydrogenase 2; RUNX1: Runt-related transcription factor 1; NPM1: nucleophosmin 1; ASXL1: additional sex combs like 1; BCOR: BCL6 corepressor; NRAS: neuroblastoma RAS viral oncogene; TET2: Tet methylcytosine dioxygenase 2; SRSF2: serine and arginine rich splicing factor 2; BCORL1: BCL6 corepressor-like 1; RAD21: RAD21 cohesin complex component; KMT2A-PTD: lysine methyltransferase 2A partial tandem duplication; ATRX: ATRX chromatin remodeler; PTPN11: protein tyrosine phosphatase non-receptor type 11; U2AF2: U2 small nuclear RNA auxiliary factor 2; NF1: neurofibromin 1; SMC1A: structural maintenance of chromosomes 1A; KRAS: kirsten rat sarcoma viral oncogene;

Suppl. Table 4 | AML patient characteristics (CAR-T cytotoxicity cohort)

BM Sample	Age	Sex	Disease Status	Molecular biology	Cytogenetics	Blasts (%)	Secondary AML
AMLBM119	59	female	Primary diagnosis	ASXL1, CSF3R, PHF6, RUNX1, SRSF2	--	>90%	Yes
AMLBM133	43	male	Primary diagnosis	NPM1, DNMT3A, NRAS	46,XY	>90%	No
AMLBM139	67	male	Primary diagnosis	ASXL1, IDH2, RUNX1, SRSF2, STAG2, NRAS, PHF6 , PRPF8 , ZRSR2	complex karyotype	10-20%	Yes
AMLBM157	50	female	Primary diagnosis	ASXL2, SMC3, FLT3-TKD	complex karyotype	50%	No

DNMT3A: DNA methyltransferase 3 alpha; IDH2: isocitrate dehydrogenase 2; RUNX1: Runt-related transcription factor 1; FLT3-TKD: Fms-like tyrosine kinase 3 – tyrosine kinase domain mutation; NPM1: nucleophosmin 1; ASXL1/2: additional sex combs like 1/2; NRAS: neuroblastoma RAS viral oncogene;; PHF6: plant homeodomain (PHD) finger protein 6; SRSF2: serine and arginine rich splicing factor 2; PRPF8: pre-mRNA-processing-splicing factor 8; ZRSR2: zinc finger CCCH-type RNA splicing factor 2; SMC3: structural maintenance of chromosomes 3; CSF3R: colony stimulating factor 3; STAG2: Stromal Antigen 2 cohesin complex component;

Suppl. Table 5 | Functional relevance of the identified targets based on DepMap CRISPR–Cas9 dependency data.

Gene	Number of evaluable models	Median Chronos score	Dependent models
PTPRG	31	−0.0917	0/31
NECTIN2	31	−0.0298	0/31
ENG (CD105)	31	−0.0156	0/31
CD33	31	0.0456	0/31
FLT3	31	−0.1162	5/31
IL3RA (CD123)	6	0.1650	0/6

CRISPR–Cas9 dependency data from the DepMap Public 26Q1 release were used to assess the functional relevance of PTPRG, NECTIN2, and ENG (CD105) [36, 37]. CD33, FLT3, and IL3RA (CD123), which are established or commonly investigated therapeutic targets in AML, were also analyzed for comparison. The Chronos gene-effect score estimates the effect of CRISPR-mediated gene knockout on cellular fitness, with scores near 0 indicating little or no effect and more negative scores indicating a greater reduction in cell proliferation or survival. Gene dependency probability estimates the likelihood that a cell-line model is dependent on a given gene. Models with a gene dependency probability greater than 0.5 were classified as dependent. Chronos gene-effect scores and gene dependency probabilities were available for 31 AML cell-line models for all genes except IL3RA, for which data were available for only six models.

Suppl. Table 6 | Primer sequences.

Forward primers (Phosphorylated)

Name	Sequence
IgHJ24	GGCCCAGGCACCCTGGTCA
IgHJvar	GGCCCGGGCACCCTGGTCA
IgHJ35	GGCCAGGGCACCCTGGTCA
IgHJ6	GGCCCAGGGACCCTCGTCA
HJ104	GGCCCTGGGACCCTCGTCA
HJ126	GGCCCGGGGACCCTCGTCA
HJ1767	GGCCCAGGAACCCTCGTCA
HJ2104	GGCCCAGGCGGCCTGGTCA
HJ2319	GGCCCAGGCACCCTCGTCA

Reverse primers (Primers used for amplification of the 28 Kasumi-1 binders listed in Fig. 2 are shown in bold.)

Name	Sequence
Kas-8 rev	CCACAAGTCATAACCACCATAAGTCCCTCT
**Kas-11 rev**	CCACAAGTCAAAGTAGTAGGAGCCACCA
**Kas-17 rev**	CCAGATATCAAAAGCATAACCATAATTCTCTCTGACA
**Kas-28 rev**	CCAGGGATCGAAAGCCCCATATAAGGA
Kas-35 rev	CCAGATCGAATAATCAGCACTACTACCACTATATT
Kas-36 rev	CCAGACCGAACCAGTATCTCTGGCA
Kas-37 rev	CCAGATGTGGCTATACCAATTATTACCACCAC
Kas-47 rev	CCACAAGTCCATACCAATAGTATAACCACCATAT
Kas-49 rev	CCAGATGTCCCCAACACTATAGCCAG
**Kas-64 rev**	CCAGATGTCATAAGCACGCCCATCACTATA
Kas-71 rev	CCACAAGTCCCCTATAACCCCTCTCA
Kas-114 rev	CCACAAGTCAAGAGTCCCAGCATCTC
Kas-119 rev	CCACAAGTTACCAGCATTATAAGCAGCCCT
**Kas-130 rev**	CCACATGTTAAGGTCAATACTATCAGCACGAAC
Kas-131 rev	CCAGAGGTCCATGCCGTAGTAATCCT
Kas-150 rev	CCACAAGTCATAACTATTACCAGTACCATATCTG
Kas-153 rev	CCAGACATCAAAAGCCATCCAGCCC
Kas-176 rev	CCAGATGTCAATTAAAAGGCCTCTGGCACA
Kas-188 rev	CCACAAATTAAACCCAGTAGTATAAGTAGCAAATCTG
**Kas-240 rev**	CCAGATGCTCATACCATTACCATAATCGCCT
Kas-297 rev	CCAGATGTTCAAGGCATAGGCATAATTATCACT
**Kas-309 rev**	CCAAATGTCAAAGTAGGAATCATCATCTCTCACA
Kas-322 rev	CCAGGGATCAAATCCATCCCAACCAC
Kas-325 rev	CCAGATGTCAAAGAACGCATAGTCATCGTCTT
Kas-350 rev	CCACAGATTAAACCCATCACCAAAAGTTCTGG
Kas-354 rev	CCACAAGTTACTATACCAACCACTACCTCTC
Kas-389 rev	CCACAAGTCGCCGATATTATAACCACTCC
**Kas-404 rev**	CCAGATGTCTAGAATCAGGATATTTCTCGCACA
**Kas-484 rev**	CCACAAGTTAGGACCCCAGCCAAAAC
Kas-485 rev	CCAGGGATCAAAAACATTACCATCACTACTACTT
Kas-495 rev	CCAGAGGTCCATGGCTCCCCA
Kas-568 rev	CCAGATGTTAGGGGGCCAATTTCTGG
Kas-616 rev	CCACAAGTCAAAGGCAGTATAATGTATACTACCA
Kas-649 rev	CCAGATGTCCATAGCAGCACCATAATATTCTCT
Kas-650 rev	CCAGATGTCAAAAGCAACACCACCCCATATTT
Kas-670 rev	CCACAAGTTCACACCCCAGCCAC
Kas-710 rev	CCAGGGATCGAAAGCCTCAACATAACCATAA
Kas-739 rev	CCAGATGGCAAAGGCATCACGATCAC
Kas-833 rev	CCAGGGATCGAAAGCACCAGTACG
Kas-856 rev	CCACAAGTCCACACCATCAACATAAGCAC
**Kas-890 rev**	CCAGGGATGATAGGCATATCCACTATACTCT
Kas-1068 rev	CCAGGAATCAAAAGCATTACCATAACCAGCAC
**Kas-1187 rev**	CCAGATGTTAAAATCGATAGTTTCACTACCATAATCCT
Kas-1203 rev	CCACAAGTCAAAGGCGGCACCAG
Kas-1236 rev	CCAGACGTCAAAGACCCAGCCATC
Kas-1337 rev	CCAGAAGGTAAACCAACTACCACTGACACA
Kas-1369 rev	CCACAAGTTAATCGTACTATCACCAACATTAAGTCT
Kas-1379 rev	CCACAAGTCCGTAGCATCAATAGCACCAA
Kas-1413 rev	CCAGACACCACCTCCAAGTATGTCTTTC
Kas-1425 rev	CCAGATGTTATGGGCACGGGCC
Kas-1454 rev	CCACAAGTCAAAACTACTAACACCACCATATCC
Kas-1544 rev	CCAGATGTCCCAGTCCCAAGAACCA
Kas-1610 rev	CCACAAGTTAAAGATATCCCAGCCACCAC
Kas-1613 rev	CCAGGGATCAAAAGCAGCACCCCA
Kas-1635 rev	CCAGATGTCAAGGCCCTCATAACCATAAC
Kas-1637 rev	CCAGATGCTATAATAACTACTGCCACTAGTTCT
Kas-1652 rev	CCACAATTCCATATAACCGCCTCTGGCA
Kas-1674 rev	CCACAAGTTAAAAGCATTAGTATAACCAGCATGTCT
Kas-1688 rev	CCACAAGTCGGCACTACCAGCAAGAA
**Kas-1707 rev**	CCAGATGTTAAAAACAGCCCAACCAATCCCT
Kas-1721 rev	CCAGATGTCAAACCCACTCCAGCCA
Kas-1773 rev	CCAGATGTCAAAGTACCATGACAGCCACT
Kas-1785 rev	CCACAAGTCATTATAATAACTGGCACTAGCCTC
Kas-1848 rev	CCAGATGGCCAAACCAGCATCACCA
Kas-1892 rev	CCACAAATTAAATGAAGTACTATAACTACCATATCTGGCA
Kas-1905 rev	CCATATGCCCCAACTACCATCACCATTATATCT
**Kas-1955 rev**	CCAGATGTTAAAGGCACCACTACTATAAGCAG
Kas-1968 rev	CCAGAAGTCATAAGTCTCATCATCAGCATTCCA
Kas-1974 rev	CCACAAGCCACTAGTAGCAGCATGAC
Kas-1975 rev	CCAGGAACTGAAACCATCCCAGCCT
Kas-1988 rev	CCACAAGTTAAAGCCGGAACTATGTCTGCTA
Kas-2042 rev	CCACAAGTTAAAGGAGGACCACTCGGTT
Kas-2043 rev	CCACAAGTCCGAACTACTACCACCATAAC
Kas-2169 rev	CCACAACCTAAGCTCATTACTCCCCCAT
Kas-2174 rev	CCAGAGGTCCATGCCGCCG
Kas-2196 rev	CCAGATGTTGAGGCCATAGGCATCATCAT
Kas-2204 rev	CCACAAGTCAAAGGCGTATCCATAATGCC
Kas-2247 rev	CCAGCCGTAAAGGCCCCAGC
**Kas-2282 rev**	CCACAAGTTATAACCACCATACGATTCTCTGG
Kas-2343 rev	CCAGATGTCAATAGCATTAGCACCACCAC
Kas-2381 rev	CCAGATGTTAGGCGAGGCACAGAAATAG
**Kas-2385 rev**	CCAGAAGTTAAAGTAGGTACCAGTATTATAACTAGCATAA
**Kas-2388 rev**	CCAGATACTAAAATACAAACCACTACTACTACTCCA
Kas-2427 rev	CCAGATGTCGAAACCCCAAGTCTCTCT
**Kas-2430 rev**	CCACAAGTTCAATTGCATGGAACCACCACTA
Kas-2455 rev	CCACAAGTCATAACCATTATTATGTCTGGCACA
Kas-2506 rev	CCACAAGTCAAGGGATAACCATCCTCTG
Kas-2536 rev	CCACAAGTCATGTCCATCACCACCATAAACA
Kas-2606 rev	CCAGATGCTAAAGTAGGACCAGCCC
**Kas-2653 rev**	CCACATGTTAAAGTAGGTACCAACATTATAACCAG
Kas-2765 rev	CCAGATGTCCGCGCCCCAGT
Kas-2768 rev	CCACAAGCTGTAACCACTACCACCAGA
**Kas-2810 rev**	CCAGATGTTTTCGATACCACTACTCCAACCAA
Kas-2817 rev	CCACAAGGTACCACTGCTACTACCAACAA
Kas-2823 rev	CCACAAGTCTAATCCATTATTACGTCTGGCACA
Kas-2826 rev	CCAGATGTTGGCACCAGCACTGTAACT
Kas-2828 rev	CCAGATGTTAAAAGGGCCACTACCTCTG
Kas-2829 rev	CCAGATGTTATAACCATTACCACCACCATATCT
Kas-2835 rev	CCAGATGTCGGAACCATCACCAACC
Kas-2837 rev	CCAGATGTCAAGAACACCAGCACCATTAATATC
Kas-2843 rev	CCATATGTCAAGGGACCAATCACTGTCC
Kas-2853 rev	CCACAAGTCATAACCACCATAATTCCCTCTG
**Kas-2861 rev**	CCAAATGTCAAAGTAGTAACCATACCCTTCTCT
Kas-2941 rev	CCAGATGCCAATTCCACCCAGCCA
Kas-2942 rev	CCACAAGTTAAAGTAGGAGGCAGTACTCCAAT
Kas-2946 rev	CCAGATCCCCATCTCAGTACTATAAGTAGCT
Kas-2968 rev	CCACAAGTCAGAAGCAATACCTCTGGCA
Kas-2978 rev	CCAGATATTACCACCACTATAAGCAAGATTCCC
Kas-3005 rev	CCAGATATCATAGCCATTAGCAGCAGCTTC
Kas-3091 rev	CCACAAGTTAAAGTTGTAAAGGTAACCCCCTCT
Kas-3139 rev	CCAGGGATCGAAAGCATTCCCGTAC
Kas-3145 rev	CCAGAGGTTATCAATTGTACCAGCTTCTCTG
Kas-3147 rev	CCACAAGTCAAAGTAGTCCCAATAATTATTACCAG
Kas-3151 rev	CCACAAGGCACCATCAGCACCC
Kas-3160 rev	CCACAAGTCAAAGTAACGAGCATCAATATATCTG
Kas-3174 rev	CCAGATATCAATGTAATCACGACTACCAACATGT
Kas-3187 rev	CCAGATGTCCCCGGCATAACCTCT
Kas-3201 rev	CCAATGATCAAGAGCCCTCCCCC
**Kas-3208 rev**	CCAGATCCCGGTATCAACATTATAATCATCTCT
Kas-1389 rev	CCAAATGTCAAGATTAAAAGCATCACCCAATCTC
Kas-2895 rev	CCACAAATTAAAAGGTTCACCAGTCCAACCAC
Kas-243 rev	CCAGAGATCCAACCGAACCACCC
Kas-358 rev	CCAGAGATCCAACCAGTCGAAGTCATCA
Kas-1122 rev	CCAGAGATCCAAGCGATACAAATCCCAG
Kas-1349 rev	CCAGGGATCCAACCGAGCATAATCACTAT
Kas-1543 rev	CCAGAGATCCAACCAGGCCCATC
Kas-3210 rev	CCAGAGATCCAACCCGTTTCTCCATCT
Kas-6 rev	CCAGAGGTCCATGTTGTAGTAAAAGTCCC
Kas-12 rev	CCAGAGGTCCATGCCGTAGTAAGC
Kas-25 rev	CCAGAGGTCCATGCCGTAGTAATCTCT
**Kas-43 rev**	CCACAAGTCCATACCAGTCCCATAATCAC
**Kas-44 rev**	CCAGAGGTCCATGCCGTAGTAAGTATAACTAT
**Kas-95 rev**	CCAGATGTCCATGCCATAGGGATCATCA
Kas-138 rev	CCAGAGGTCCATGCCGTACCAATCT
Kas-158 rev	CCAGAGGTCCATGCCGTAATACCCA
Kas-173 rev	CCAGAGGTCCATGCCGTAGTAATAACCATAA
Kas-205 rev	CCAGGGGTCCATGCCGTACCAT
Kas-214 rev	CCAGAGGTCCATGCCGTAGTAAGTACTT
Kas-225 rev	CCAGATAACATCACTATAATTAGGTAGGCCTCT
Kas-268 rev	CCAGAGGTCCATGCCGTAGTCAC
**Kas-277 rev**	CCAGAGGTCCATGCCAATACTATAAGCAG
Kas-323 rev	CCAGAGGTCCATGCCGTAGTAATTAGTAATATA
Kas-326 rev	CCAGGGGTCCATGCCGTAGG
Kas-344 rev	CCAGAGGTCCATGCCGTAGTACCAA
Kas-642 rev	CCAGGGGTCCATGCCATCACCATAA
**Kas-683 rev**	CCAGGAGTCCATGGCGTAGGAAAAATAATCAT
Kas-796 rev	CCAGAGGTCCATGCCATAATAGCCATAAC
Kas-931 rev	CCAGGGGTCCATGCCGTAGACA
Kas-1047 rev	CCAGGGGTCCATGCCCCAG
Kas-1062 rev	CCAGGGGTCCATGGCGCCG
Kas-1064 rev	CCAGGGGTCCATGCCGTAGTAATCA
Kas-1096 rev	CCAGGGGTCCATGCCGTAACCATAT
Kas-1211 rev	CCAGAGGTCCATGCCGTAGGTAAC
Kas-1251 rev	CCAGAGGTCCATGCCGTAGTAGG
Kas-1312 rev	CCAGAGGTCCATGGTTACTAACTTTCTAGCA
Kas-1321 rev	CCAGAGGTCCATGCCGTAGGAAAGA
Kas-1455 rev	CCAGAGGTCCATGCCGTAGTATCC
Kas-1466 rev	CCAGATGTCCACACCACCATCATAACCA
Kas-1475 rev	CCAGAGGTCCATGCCATAAAAGGTATCATCT
Kas-1585 rev	CCAGAAGTCCATGCCGTAATAATCTCTCG
Kas-1725 rev	CCAGAGGTCCATGCCGTAATAACCAC
Kas-1922 rev	CCAGGGGTCTATGCCGTAGTACCAT
**Kas-1948 rev**	CCAGAGGTCCATGCCGTAATCGAAAC
Kas-1983 rev	CCAGAGGTCCAAGCCGTAGCC
Kas-2010 rev	CCAGAGGTCCATGACGTAGTAATGTCTG
Kas-2067 rev	CCAGATACCATCACTATAATTAGGTAGGCCTCT
Kas-2324 rev	CCACAAATTAAAGGAACCACTAATACCATATCTGG
Kas-2414 rev	CCAGAGGTCCATGTCGTAGTCGTCT
Kas-2450 rev	CCAGAGGTCCATGCCGTAGTAACTTCT
Kas-2526 rev	CCAGAGGTCCATGCCGTAGCC
Kas-2583 rev	CCAGAGATTATACCCGTACCAATAAGCAGTACT
Kas-2604 rev	CCAGGGGTCCATGGCGTAGTAATAG
Kas-2745 rev	CCAGAGGTCCATGCTGTAGTAAAAGTCC
Kas-2770 rev	CCAGGGGTCCATGCCGTAGTAAG
Kas-2801 rev	CCAGGGGTCCATGCCGACGAA
Kas-2983 rev	CCAGAGGTCCATGCCGTAGTAACCA
Kas-2996 rev	CCAGAGGTCCATGCCGCCATATGA
Kas-3102 rev	CCAGGGGTCCATGCCGTAAGCA
Kas-3118 rev	CCAGAGGTCCATGTTGTAGTAAAAGTCCC
Kas-3135 rev	CCAGAGGTCCATGCCGTAGGAATAACTA
Kas-3179 rev	CCAGAGGTCCAAGCCATAACTGTAAGAAGTA
Kas-104 rev	CCAGATGTCATTACTACTCCAACCAGGATC
Kas-126 rev	CCAGAGGTCCATGCCGTAGGCATA
Kas-1767 rev	CCAGAGGTACATGTCGTCGTAATAATGACCA
Kas-2104 rev	CCAGATGGTAGCGCTACCACTTTCCT
Kas-2319 rev	CCACAAGTTAAAGGTAGCATAACCATCATAAGCAT
Kas-2677 rev	CCAAATGTCATAATAGGCAGTAACACCACTATATTTC

Supplementary Figures

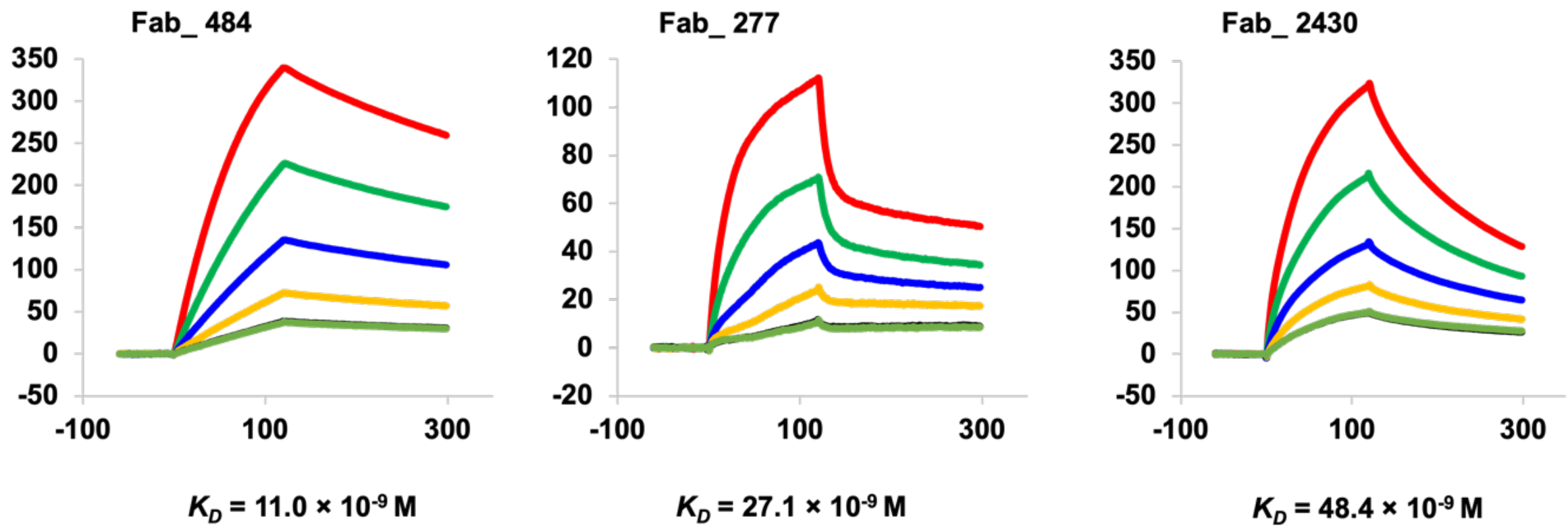

Suppl. Fig. 1 | SPR sensorgrams showing the affinity of Fab_484 (left panel, $K_D = 11.0 \times 10^{-9}$ M), Fab_277 (middle panel, $K_D = 27.1 \times 10^{-9}$ M), and Fab_2430 (right panel, $K_D = 48.4 \times 10^{-9}$ M) to human Nectin-2.

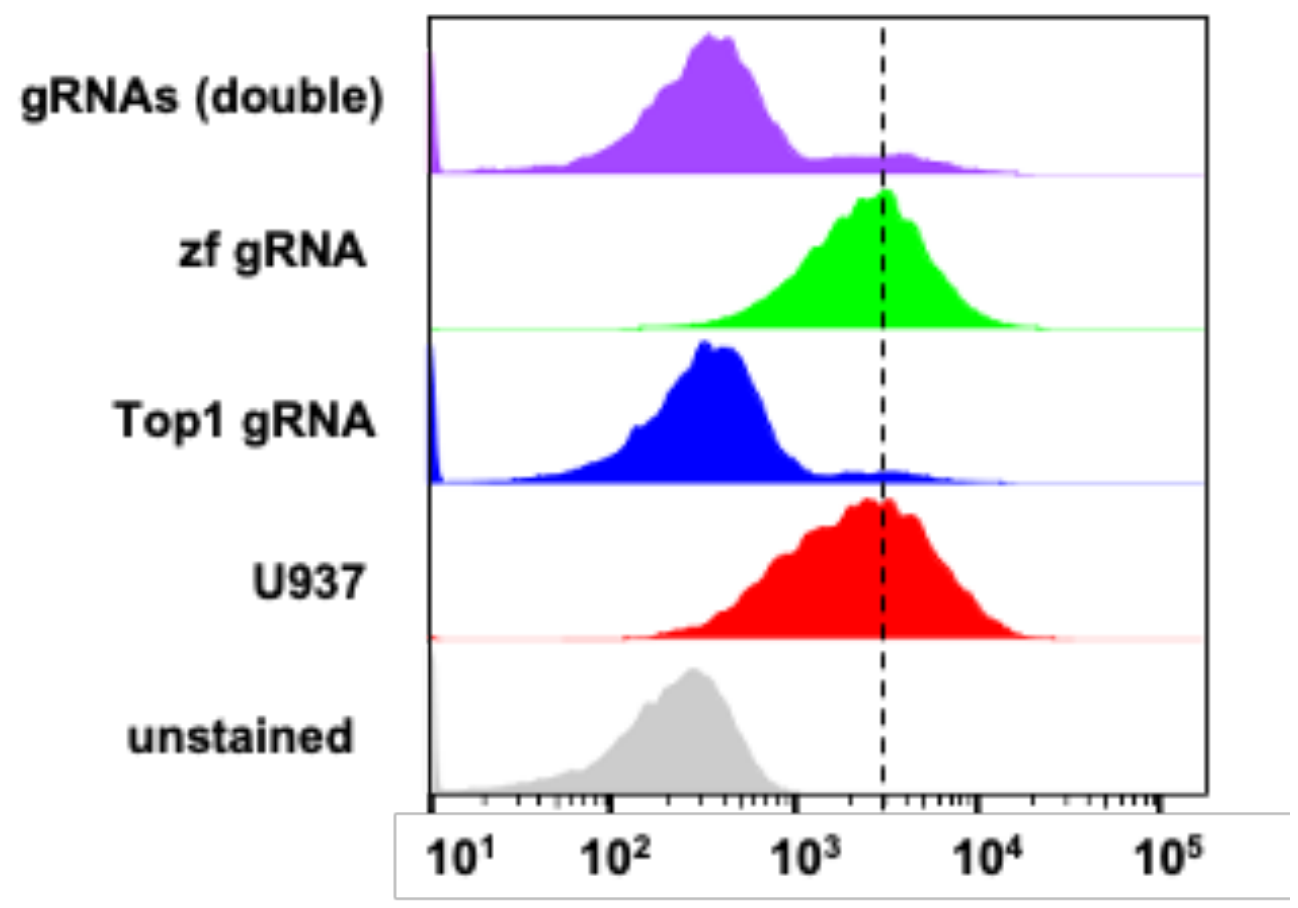

Suppl. Fig. 2 | Flow cytometry histograms of the binding of a commercial anti-CD105 mAb (clone 43A3, Alexa 647-labeled) to U937 cells. Blue; cells transduced with two CD105 gRNAs (Top1 and zf gRNAs). Cyan; cells transduced with CD105 zf gRNA. Green; cells transduced with CD105 Top1 gRNA. Red; parental U937 cells. Gray; unstained U937. These results suggested that the knockout using the zf gRNA was unsuccessful.

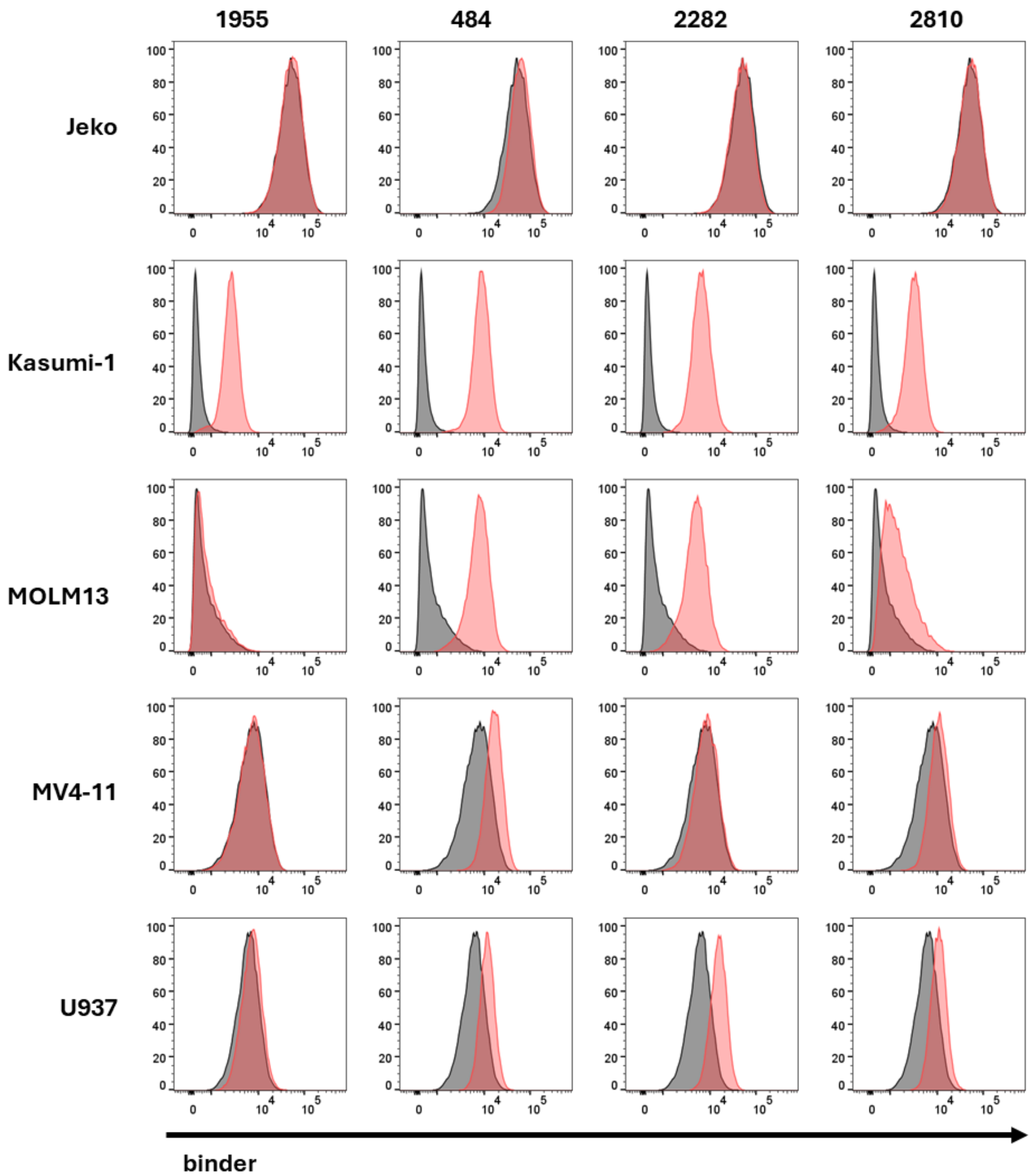

Suppl. Fig. 3 | Flow cytometry histograms of AML cell lines stained with IgG1s (LALAPG) 1955 (anti-PTPRG), 484 (anti-Nectin-2), 2282 (anti-Nectin-2), and 2810 (anti-CD105). Histograms show staining with IgG1 binders and secondary antibody (red) and the secondary antibody only control (gray).

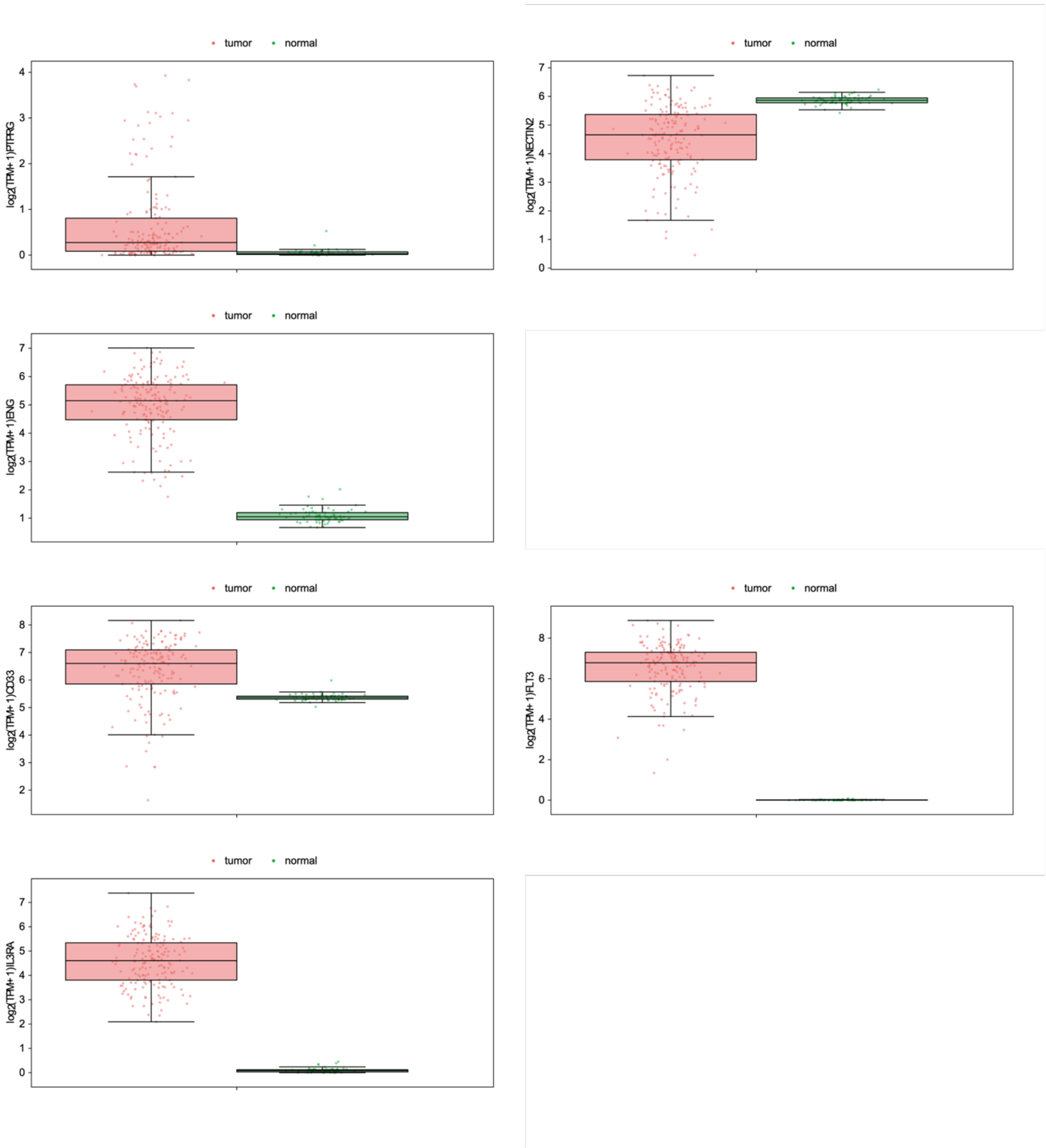

Suppl. Fig. 4 | Transcript-level expression analysis using the GEPIA3 box plot module. The expression levels of PTPRG, NECTIN2, and ENG (CD105) in AML were compared with those in normal samples. CD33, FLT3, and IL3RA (CD123), which are commonly investigated therapeutic targets in AML, were also analyzed for comparison. TCGA-LAML tumor samples are shown as red, whereas normal samples from the Genotype-Tissue Expression (GTEx) project are shown in green. Expression values are presented as log2(TPM + 1), where TPM denotes transcripts per million. The "LAML Tumor" dataset and the "TCGA paired peritumor + GTEx normal" option were selected as the tumor dataset and normal reference, respectively. The plots were generated using GEPIA3 [35].

A

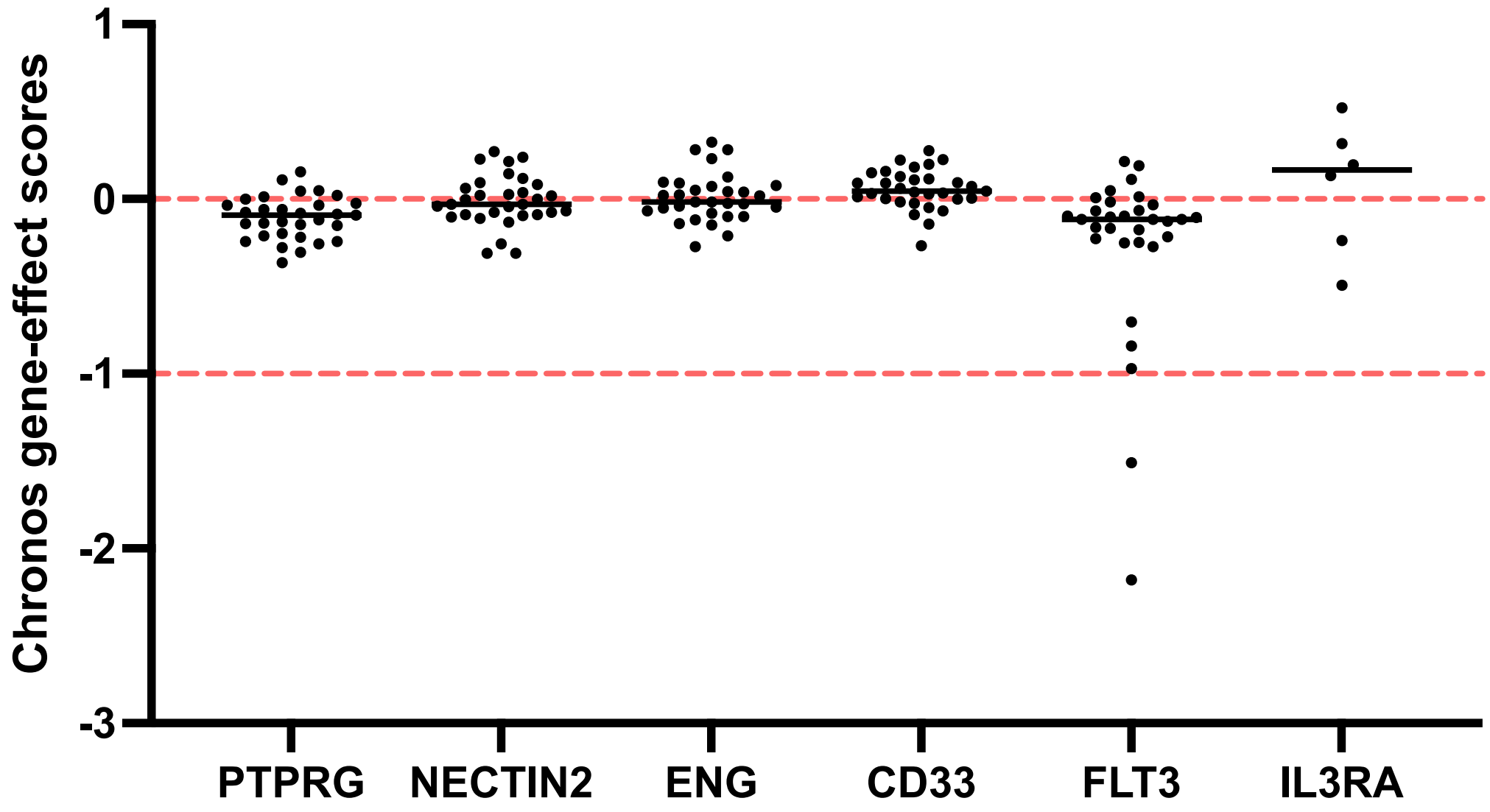

B

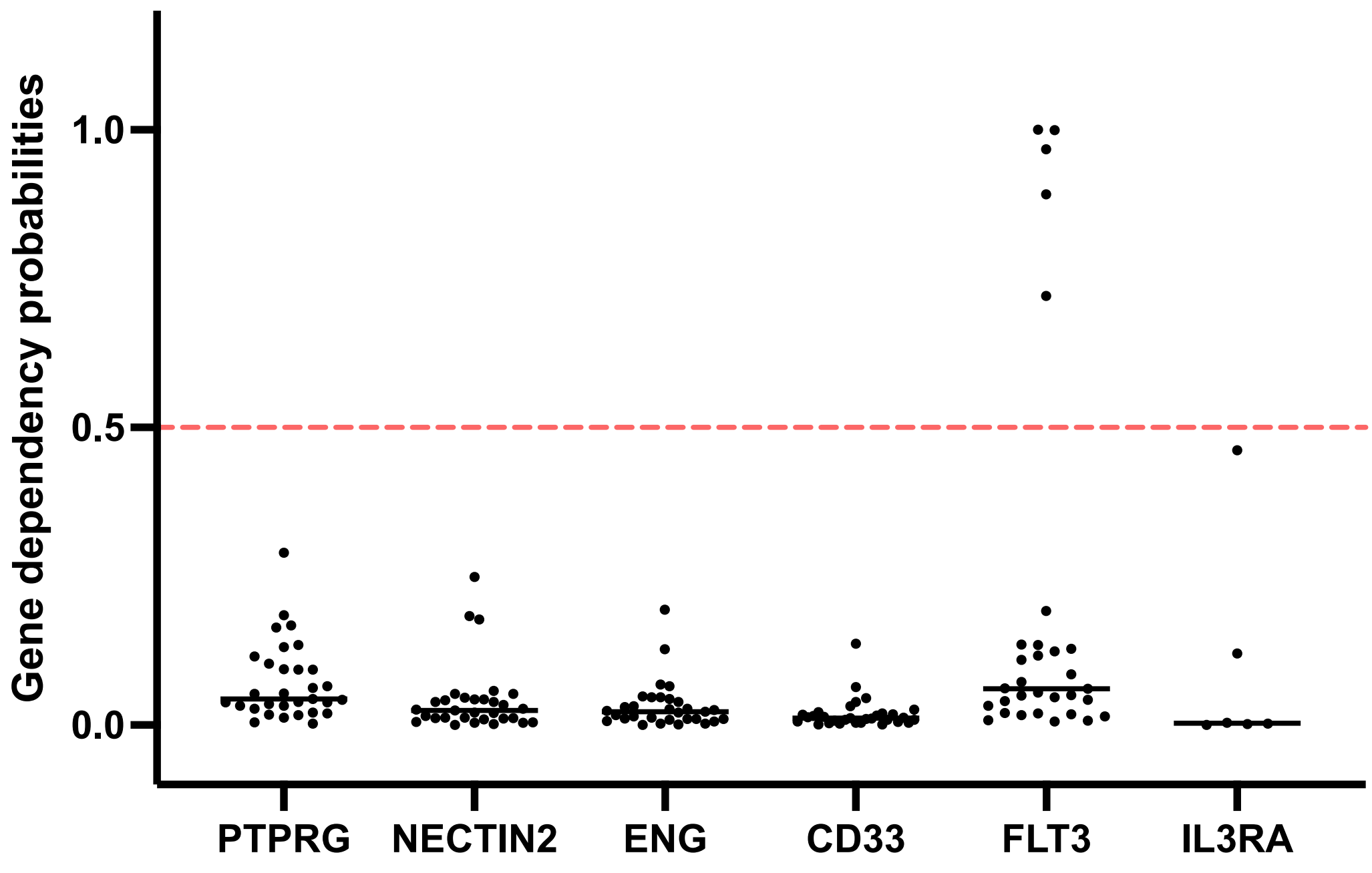

Suppl. Fig. 5 | Functional relevance of the identified targets based on DepMap CRISPR–Cas9 dependency data [36, 37]. (A) Chronos gene-effect scores for PTPRG, NECTIN2, ENG (CD105), CD33, FLT3, and IL3RA (CD123) across AML cell-line models from the DepMap Public 26Q1 release. Each dot represents one AML model, and horizontal bars indicate the median value for each gene. Chronos scores estimate the effect of CRISPR-mediated gene knockout on cellular fitness, with scores near 0 indicating little or no effect and more negative scores indicating a greater reduction in cell proliferation or survival. The line at −1 indicates the approximate median effect of common essential genes. (B) Gene dependency probabilities for the same genes across AML cell-line models. Each dot represents one AML model, and horizontal bars indicate the median value for each gene. Models with a gene dependency probability greater than 0.5 were classified as dependent as summarized in Suppl. Table 5. The red dashed line indicates the dependency threshold. Data were available for 31 AML cell-line models for all genes except IL3RA, for which data were available for only six models.
